# Stateful-Service-Based Pupil Recognition in Natural Light Environments

**DOI:** 10.3390/healthcare10050789

**Published:** 2022-04-23

**Authors:** Rih-Shen Ke, Gwo-Jiun Horng, Kuo-Tai Chen, Kuo-Chang Lee

**Affiliations:** 1 Department of Computer Science and Information Engineering, Southern Taiwan University of Science and Technology, Tainan 71005, Taiwan; kerkershownow@gmail.com; 2 Chi Mei Hospital, Tainan 71005, Taiwan; 890502@mail.chimei.org.tw (K.-T.C.); iamgord@hotmail.com (K.-C.L.)

**Keywords:** hue saturation value, adaptive threshold, low-pass filtering, morphology, contour inspection

## Abstract

Smartphones are currently extensively used worldwide, and advances in hardware quality have engendered improvements in smartphone image quality, which is occasionally comparable to the quality of medical imaging systems. This paper proposes two algorithms for pupil recognition: a stateful-service-based pupil recognition mechanism and color component low-pass filtering algorithm. The PRSSM algorithm can determine pupil diameters in images captured in indoor natural light environments, and the CCLPF algorithm can determine pupil diameters in those captured outdoors under sunlight. The PRSSM algorithm converts RGB colors into the hue saturation value color space and performs adaptive thresholding, morphological operations, and contour detection for effectively analyzing the diameter of the pupil. The CCLPF algorithm derives the average matrix for the red components of eye images captured in outdoor environments. It also performs low-pass filtering, morphological and contour detection operations, and rule-of-thumb correction. This algorithm can effectively analyze pupil diameter in outdoor natural light. Traditional ruler-based measurements of pupil diameter were used as the reference to verify the accuracy of the PRSSM and CCLPF algorithms and to compare their accuracy with that of the other algorithm. The errors in pupil diameter data were smaller for the PRSSM and CCLPF algorithms than for the other algorithm.

## 1. Introduction

Physicians often use advanced medical devices for pupil examination to identify a patient’s condition. Pupil examination devices are expensive and thus increase a hospital’s expenditure on medical equipment. Pupil testing is a crucial topic in the medical field. When advanced medical devices for testing pupils are unavailable, doctors can use a small medical flashlight to observe changes in the eyes of patients. The successful use of this method often requires years of knowledge and practical experience, and diagnostic results differ by physician.

Currently, smartphones are widespread worldwide, and with advances in hardware quality, the image quality of smartphone cameras is increasing. Specifically, smartphones contain high-resolution cameras and multiple applications and are equipped with high-speed internet functions to facilitate web browsing. For these reasons, smartphones have become critical devices in daily life. As smartphones have high penetration and are easy to access worldwide, in the present study, we examined whether eye images captured using smartphones can be analyzed using medical devices for pupil assessment.

**Figure 1:**
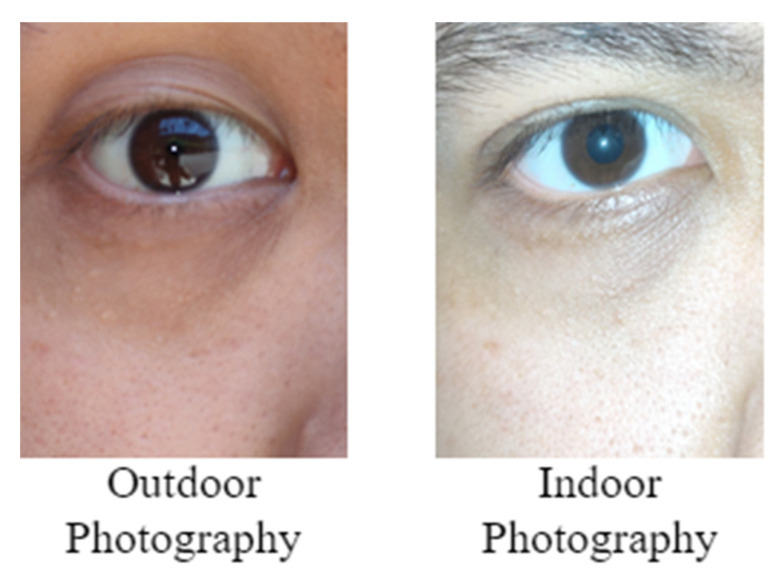
Eye images of Asian people that were captured under different light sources.

Smartphone images are presented in the RGB color space. In general, most Asian people have dark brown or black irises. Figure 1 displays Asian people’s eye images obtained in outdoor and indoor environments.

Image recognition is being increasingly used to support diagnostic procedures, and imaging technology is being incorporated into an increasing number of end devices or applications to aid tasks. In this study, we designed two analytical algorithms for analyzing pupil images that were captured under outdoor sunlight and indoor lighting for an Asian population.

The rest of this paper is organized as follows. Section 2 describes the literature related to image recognition and image analysis. Section 3 details the architecture of the developed image analysis system, which includes a pupil recognition mechanism for indoor pupil analysis and a low-pass filtering (LPF) algorithm for outdoor pupil analysis. Section 4 presents a comparison of the proposed algorithms with the other algorithm [1]. Finally, Section 5 presents the findings of this study and directions for future research.

## 2. Related Work

### 2.1. Image Recognition

With advances in hardware technology and decreases in hardware costs, the use of image recognition has increased considerably in various applications, including medical image processing, robotic analog vision, surveillance (including military surveillance), and remote sensing applications.

In image analysis, images are usually preprocessed to analyze relevant image features quickly and accurately. Image preprocessing methods include noise reduction, mathematical morphological operations, and color processing.

Noise reduction involves reducing the noise in images. Image noise reduction algorithms include Gaussian smoothing (blur) and median filtering. In addition to basic noise reduction algorithms, researchers have proposed improved noise reduction algorithms. For example, Chowdhury et al. proposed a dynamic filtering algorithm [2] that eliminates Gaussian noise in digital images by using a Gaussian kernel and Gaussian function. Lyakhov et al. proposed an adaptive median filtering algorithm for reducing image pulse noise [3]. This algorithm can effectively eliminate extreme noise from images.

### 2.2. Mathematical Morphology

Mathematical morphological operations, including image dilation, erosion, opening, filling, and closing, are often used for image preprocessing. In the dilation operation, structural elements are used to enlarge an image. If the edge pixels in the original image and the pixels of the structural element overlap, pixels are added at the center of the structural element. In this study, we used the aforementioned technique to filter out noise from eye images.

In the erosion operation, structural elements are used to reduce the number of shapes in an image. In contrast to the dilation operation, in the erosion operation, the center of the structural element traverses the image. A pixel in the original image is eroded if this pixel is located at the center of erosion and some pixels overlap the structural element.

In the opening operation, erosion is conducted first, followed by dilation. The opening operation results in the passivation of the sharp angle of the graphic extrusion. In the closing operation, dilation is conducted first, followed by erosion. This operation results in the passivation of the sharp angle inside the graphic and the filling of small holes in the image.

**Figure 2:**
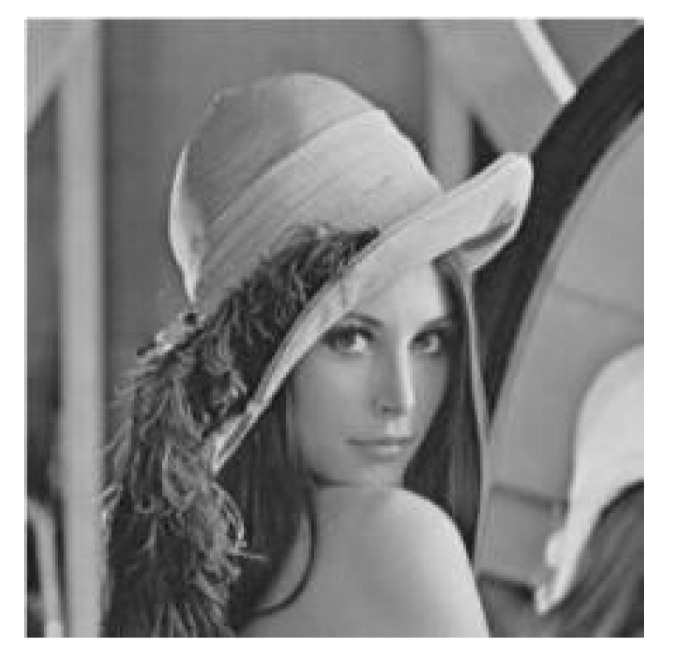
Grayscale image.

Many scholars have proposed grayscale processing algorithms (Figure 2). For example, Tian et al. proposed a grayscale projection image stabilization algorithm [4], which can improve the stability of grayscale projection images. Hasan et al. proposed a contrast enhancement scheme for deriving high grayscale contrast [5].

**Figure 3:**
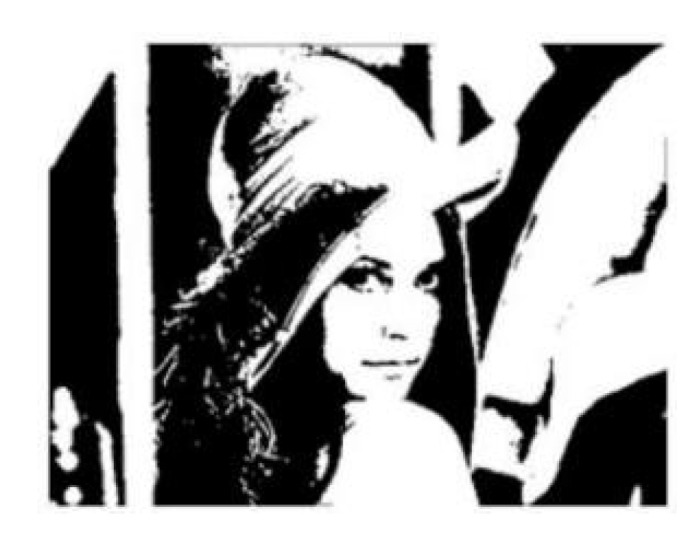
Binarized image.

Several have been conducted on binary image processing. For example, Bonteanu et al. proposed a binarization process based on adaptive thresholding [6], which can effectively determine the distribution range of pupils in human eyes. Wentao et al. proposed an optimal thresholding method [7] for effectively identifying smart cars in images. To ensure accurate routing information for smart cars, Cao et al. proposed a QR code recognition algorithm for complex and fuzzy environments [8]. In this algorithm, binary images are obtained through adaptive thresholding, and the binarized QR code is then decoded. Figure 3 displays a binarized image.

In general, an image is composed of the three primary colors: red, green, and blue. Images in the RGB color space are converted into the Hue Saturation Value (HSV) color space to obtain an increased number of image color features in order to diversify image pixels.

**Figure 4:**
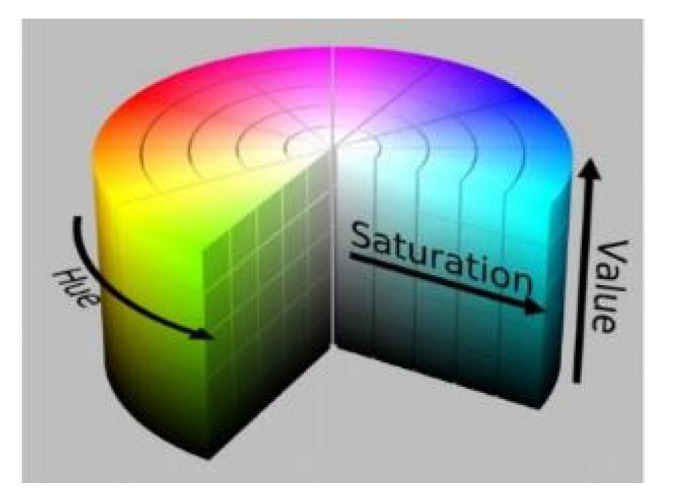
Image in the HSV color space.

Mary et al. proposed a method involving the use of histogram thresholding and the HSV color space for establishing the level of sweetness of *Muntingia calabura*, a well-known roadside fruit tree, in the Philippines [9]. Sari et al. [10] proposed an HSV color space conversion method for processing rice leaf images and determining the correct fertilizer dosage for different rice varieties. Nugroho et al. proposed an HSV saturation channel to classify *Plasmodium* as malignant or benign [11]. Figure 4 displays an image in the HSV color space. In the present study, we analyzed images of pupils captured under an indoor environment through HSV color space conversion.

### 2.3. Image Analysis

Image processing involves image adjustment processes, such as image encoding, color contrast adjustment, and noise reduction. In addition to the aforementioned processes, image analysis involves the interpretation of image content.

To explore the content of an image in detail, mathematical models and image processing techniques must be used to analyze the relationship between the underlying and upper-level information in an image. Image processing is a type of signal processing technique, and many signal processing techniques such as LPF can be used for analyzing image features. As image analysis techniques involve analyzing the relationship between the underlying and upper-level information, the rule-of-thumb principle can also be used to determine image characteristics.

Several studies have used low-pass filters in image analysis. Matsuo et al. developed an 8 K film codec preprocessing device with the functions of LPF and noise reduction [12] to improve the efficiency of film encoding. Moreover, Liu et al. proposed a frequency-domain LPF method for noise reduction in images [13]. In the present study, LPF was used to analyze the color components of the pupil in eye images captured in an outdoor environment.

The rule of thumb is a widely used principle for memorizing certain values and making decisions in various fields, including mathematics, psychology, and computer science. This rule focuses on practical applications rather than theory. In computer science, Youn et al. proposed a rule of thumb for the cross-array size of resistive random-access memory to avoid a high HRS current in read operations [14]. Gao et al. proposed a rule of thumb for evaluating deep implant antenna loss to improve radiation efficiency [15]. In the present study, the rule-of-thumb principle was used for image analysis. In [16,17,18], we have developed a fast method that can be applied to universal mobile devices and can perform and check for pupil changes in general mobile devices, since most of the users are doctors, however, in most cases it can be used in a space environment or without a network, so the deep learning method is not used in this study.

This study involved image recognition and image analysis. An image analysis system was adopted to determine the relative pupil diameter under different light sources. Image recognition is a method used for object identification, tracking, and measurement, and the term “image recognition” is closely related to the term “image analysis.” Image analysis involves exploring the data of an image to identify the features of the image and investigating the characteristics of different layers in the image.

## 3. Proposed System

**Figure 5:**
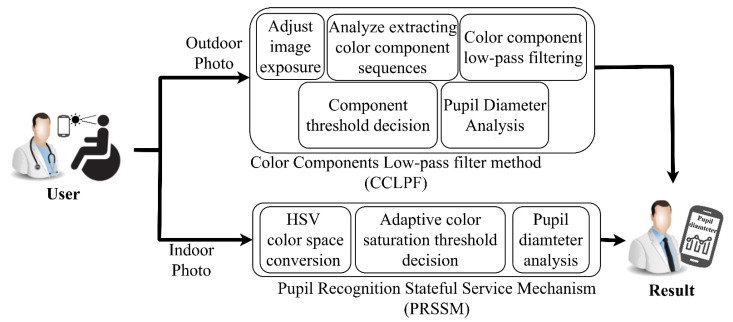
Schematic of the developed system for pupil diameter detection.

The motivation of the present study was to analyze eye images captured under different light sources for an Asian population. Such images were captured through smartphones and sent to a server for calculation and analysis. A data curve of the individuals’ pupil diameters was established to identify pupil constriction. The aim of this study was to obtain pupil diameter data for assisting physicians in diagnosing conditions. Figure 5 displays the architecture of the system developed for pupil diameter detection. This system comprises a color component LPF (CCLPF) algorithm and a stateful-service-based pupil recognition mechanism (hereafter referred to as PRSSM). Table 1 presents the abbreviations used in the system.

### 3.1. CCLPF Algorithm

The CCLPF algorithm was designed for processing 333 × 250 pixel eye images captured in outdoor sunlight in an Asian population. This algorithm uses a 3 × 3 matrix to extract data related to the red component in the RGB space after adjusting the image exposure by using the ImageEnhance function from the Python Imaging Library (PIL). Thus, a total of 82,088 data points are extracted from the red component. LPF is executed to remove the first 5000 data points belonging to the lowest color component; subsequently, the maximum and minimum values are extracted and averaged to obtain the final threshold for extracting the red color component.

After extracting pixels with a lower intensity than the final intensity threshold, morphological operations and contour detection can be used for effectively obtaining the pupil contour. Pupil correction is then used to correct the obtained pupil diameter data. After the aforementioned steps are completed, the color of a pupil captured in outdoor sunlight can be determined, and the pupil diameter curve for an individual can be obtained.

The PRSSM algorithm was designed for analyzing eye images captured for Asian individuals under indoor artificial light sources. The color differentiation between the iris and the pupil in indoor artificial light can be enhanced through HSV color space conversion. In the present study, the pupils in the captured images exhibited high saturation. Correlation, by adjusting the extraction threshold of saturation, can be well extracted from the pupil color distribution range.

We designed an adaptive thresholding method for automatically determining the optimal saturation threshold by detecting the increase in color ratio after adjusting the saturation threshold. After the color distribution range of pupils is extracted using the optimal saturation threshold, morphological opening and contour detection techniques, such as CCLPF, can be used for effectively extracting the pupil contour. Finally, pupil correction can be used to correct the pupil diameter. The PRSSM algorithm was determined to provide more accurate results than the CCLPF algorithm.

**Figure 6:**
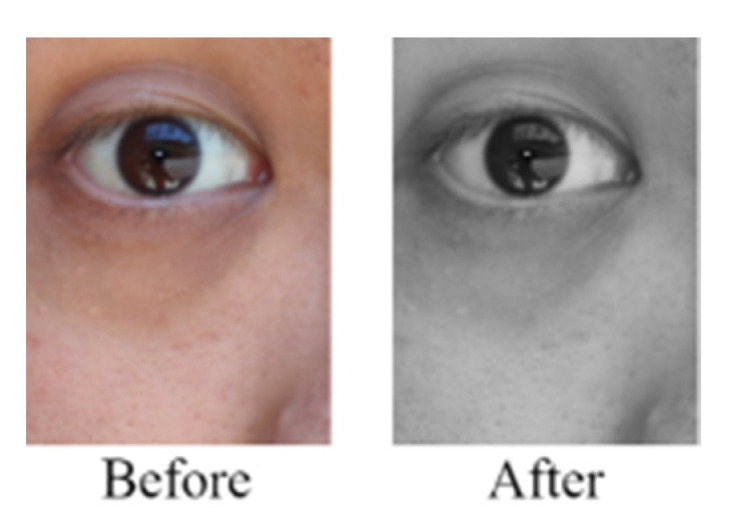
Grayscale operation on one of the captured images.

The RGB mode is currently the main mode for displaying images in electronic systems. In this study, smartphones were used for quickly capturing images of the eyes of 80 Asian individuals under sunlight. The captured images were resized to 333 × 250 pixels for analysis. As both the iris and pupil exhibit dark colors, distinguishing their color distribution ranges by using grayscale operations is difficult. Figure 6 illustrates a grayscale operation on one of the aforementioned images.

To solve the difficulty in distinguishing the color distribution ranges of the pupil and iris through grayscale operations, we analyzed the RGB tricolor channel of trilogromatic light mode. In outdoor environments, strong light factors often engender improvements in Exposure value (EV). Table 2 presents the EV in different environments.

**Figure 7:**
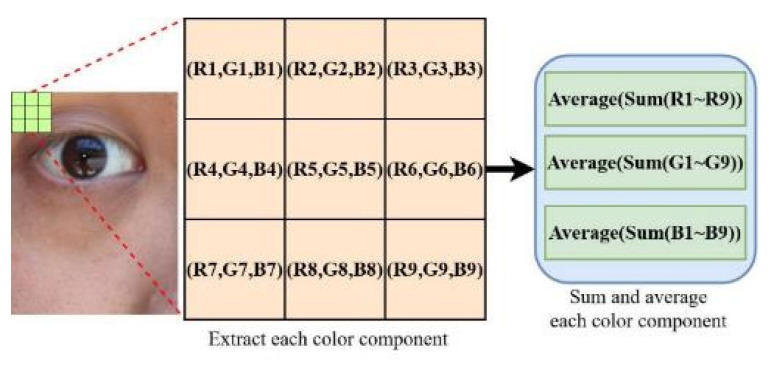
Matrix mean analysis for one of the captured images.

As presented in Table 1, the illuminance under the hot sun is higher than that in an office or a classroom by four EV. In eye images that are captured in this environment in an Asian population, the colors of the iris and pupil are extremely similar. To distinguish between the iris and the pupil in the aforementioned images, a 3 × 3 matrix was used in this study for analyzing the components of each color channel for the iris and pupil. Figure 7 illustrates the matrix mean analysis for one of the captured images.

**Figure 8:**
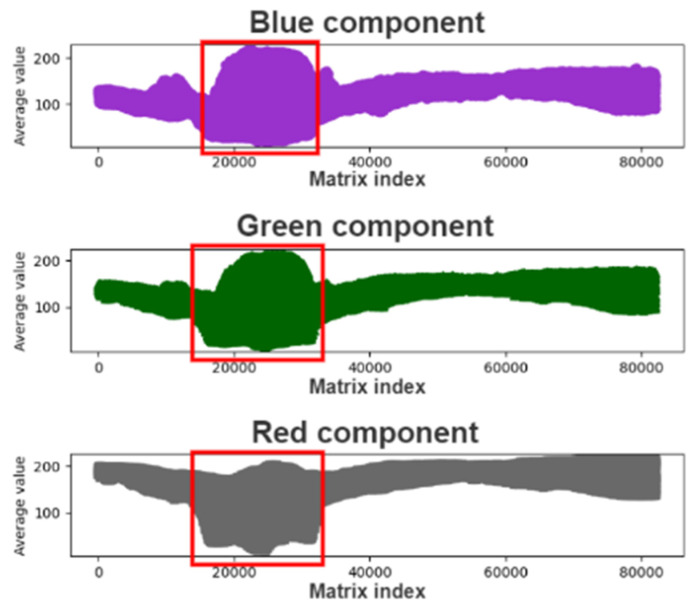
Graph for the average color component matrix.

Figure 8 displays the means of each color component matrix. As indicated in this figure, the rate of change of the color components increased in the red frame. Most of the examined pupils were black, which is represented by an intensity of zero in the red, green, and blue channels. Therefore, considering the color components with the lowest average value (i.e., the red frame, as indicated in the figure), this study examined the correlation between the RGB patterns and pupil color.

**Figure 9:**
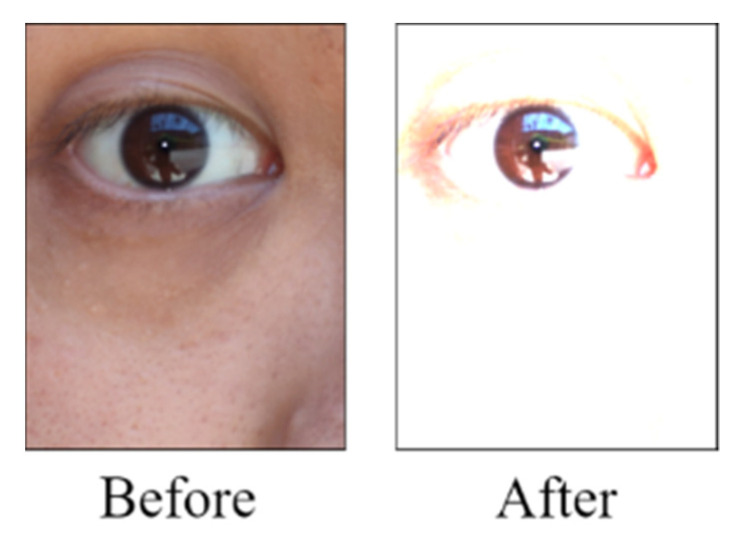
Eye image obtained after increasing the exposure.

We used the ImageEnhance function from the PIL for increasing the exposure of images and improving the color distinction between the pupil and the iris. The enhancement parameter in this function was set to three. Figure 9 depicts a comparison of the eye image obtained after increasing the exposure with an unadjusted image (i.e., Rainbow). The color difference between the iris and the pupil was effectively enhanced by increasing the exposure.

#### 3.1.1. Extraction of Color Components

**Figure 10:**
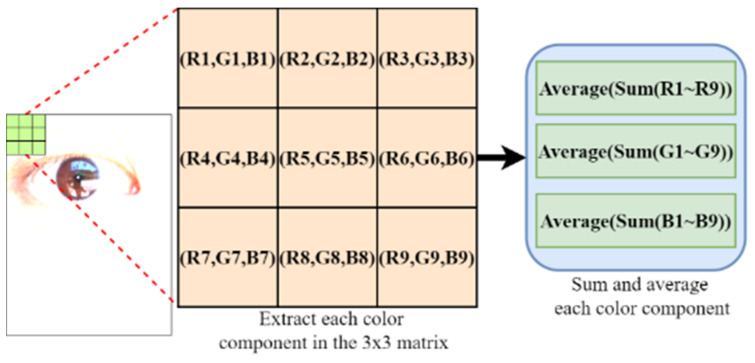
Average matrix for an image with enhanced exposure.

After increasing the exposure of the images, an average 3 × 3 matrix was applied to the image again for analysis. Figure 10 illustrates the analysis results, indicating enhanced exposure.

**Figure 11:**
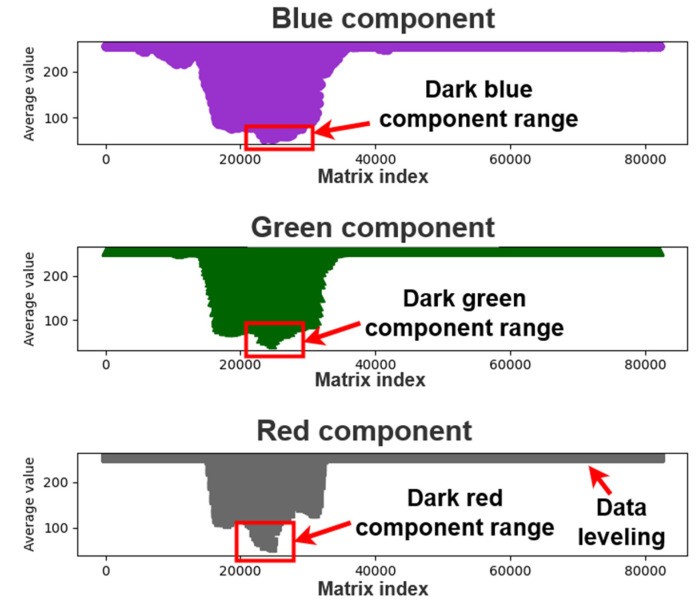
Dark component intervals for an image with enhanced exposure.

We analyzed the matrices for the images with enhanced exposure and visualized the new matrix average data. Figure 11 displays a graph of the average matrix of color components for an image with enhanced exposure. As displayed in this figure, each color component improved considerably, and the light color components were flattened. As black has an intensity of zero in all channels of the RGB color space and most Asians have black pupils, we extracted and analyzed the dark interval of each color component to find the color of the pupil. Figure 11 depicts the dark interval of each color component.

**Figure 12:**
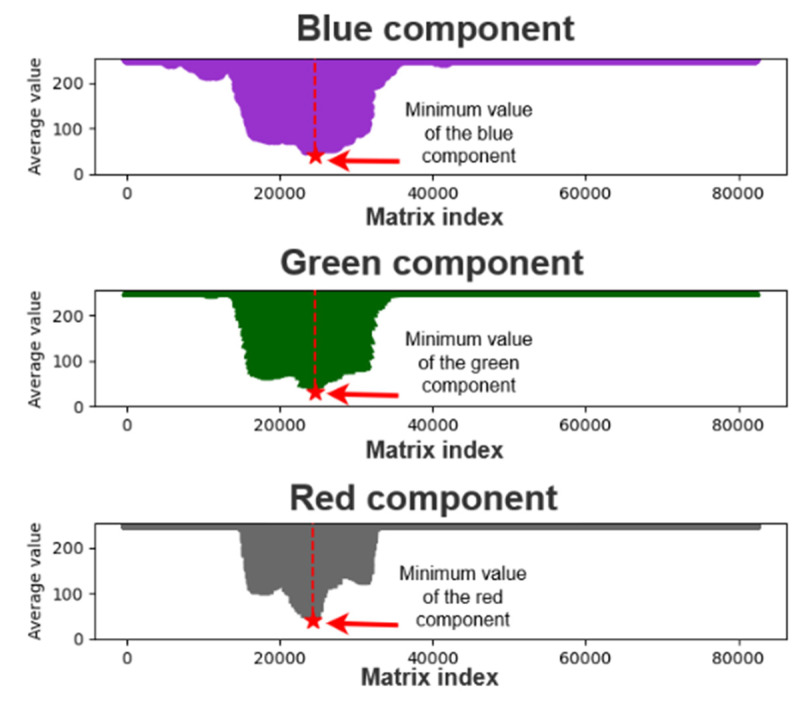
Color component minimum matrix mean index plot.

**Figure 13:**
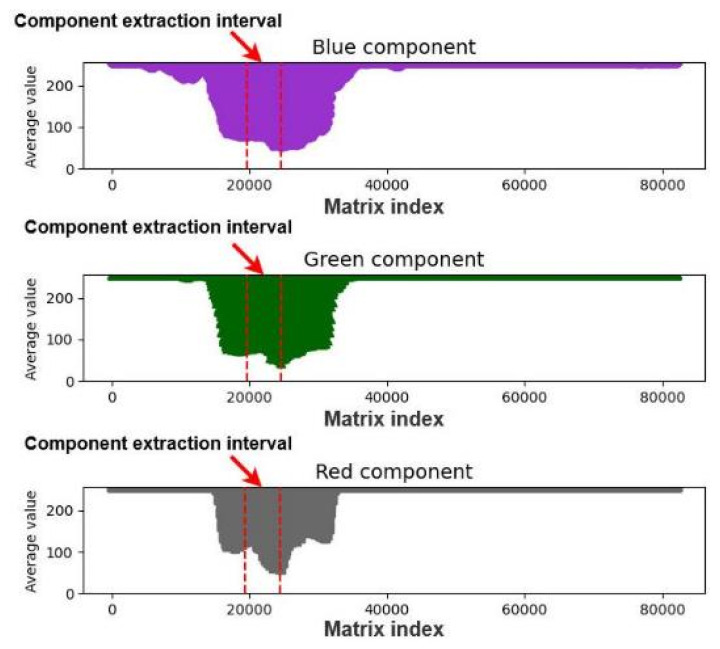
Plot of the color component extraction interval.

To find the color components of the pupil, we searched for the minimum values of each color component in the array of matrix averages; recorded the index values after determining the minimum average matrix of color components; and extracted 5000 pieces of data for analysis. Figure 12 and Figure 13 display the plots of the minimum average matrix index and color component interval, respectively.

#### 3.1.2. CCLPF Module

**Figure 14:**
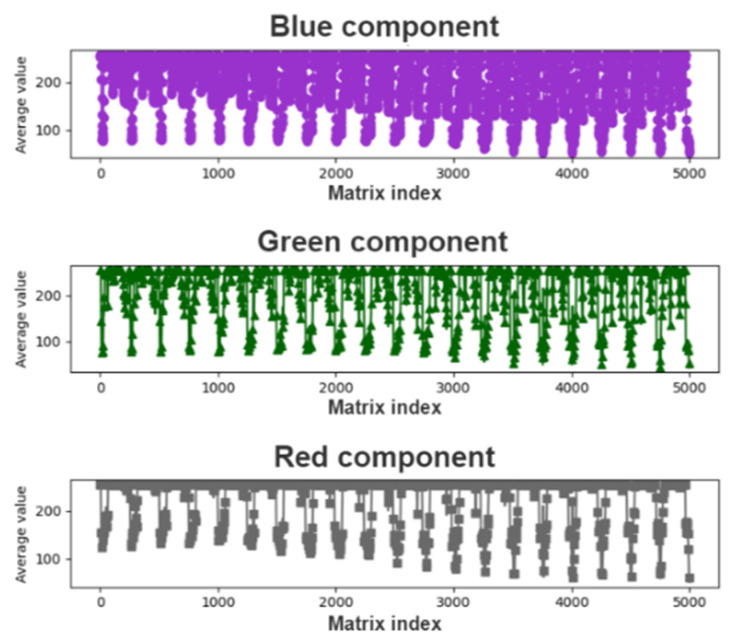
Graph of the extraction interval.

Figure 14 depicts the graph of the extraction interval, indicating considerable noise, which could cause large fluctuations in the graph. Although noise is not noticeable in the curve depicted in Figure 12, image noise can be significantly ameliorated through data subdivision.

**Figure 15:**
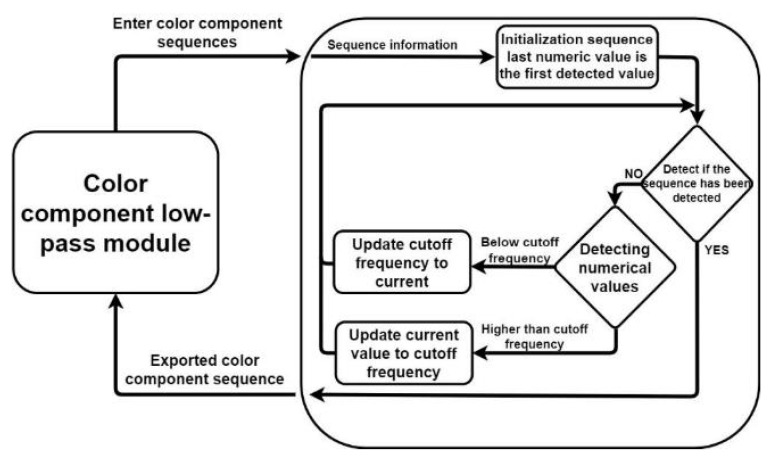
Architecture of the developed CCLPF module.

To filter out noise from the curve, LPF can be performed. A CCLPF module was designed in this study, and Figure 15 illustrates the architecture of this module.

The first value of the data series input into the CCLPF module serves as the initialization cutoff frequency. A check is performed to determine whether the entire sequence has been filtered. If the entire sequence has not been filtered, a check is performed to determine whether the values in the sequence are less than or greater than the cutoff frequency. The CCLPF module modifies values greater than the cutoff frequency to the current cutoff frequency. Moreover, if a value is less than the cutoff frequency, the CCLPF module reduces the cutoff frequency to this value.

**Figure 16:**
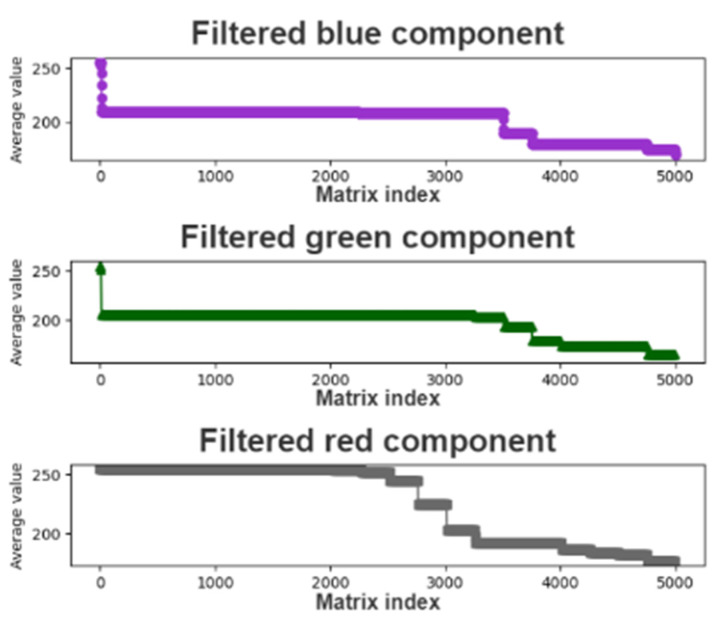
Plot of the mean color component matrix obtained after low-pass filtering.

The purpose of filtering the mean color component matrix through the CCLPF module is to analyze filtered data gradients. The matrix sequence filtered by the CCLPF module allows the overall data curve to be flatter. Figure 16 depicts the plot of the mean color component matrix obtained after LPF.

#### 3.1.3. Component Threshold Decision

**Figure 17:**
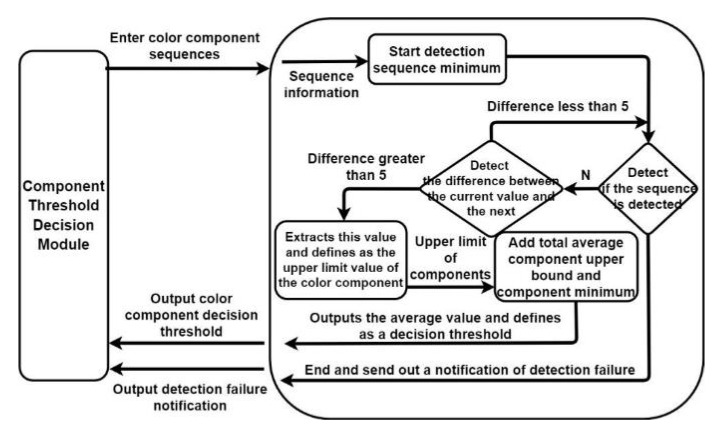
Architecture of the developed component threshold decision module.

**Figure 18:**
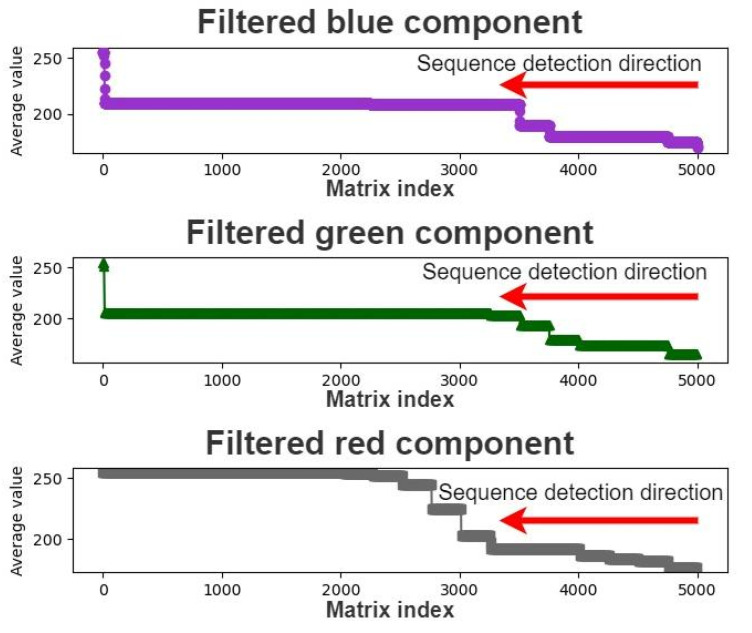
Sequence detection direction of the developed component threshold decision module.

We designed a component threshold decision module to determine optimal component thresholds for calculating color component differences. Figure 17 depicts the architecture of the developed component threshold decision module. We can analyze the correlation between the mean components of the matrix of each color channel and the pupil color by using the optimal threshold calculated by the developed component threshold decision module. This module first filters the mean color component matrix, beginning from the lowest value. Figure 18 displays the sequence detection direction of the developed component threshold decision module.

**Figure 19:**
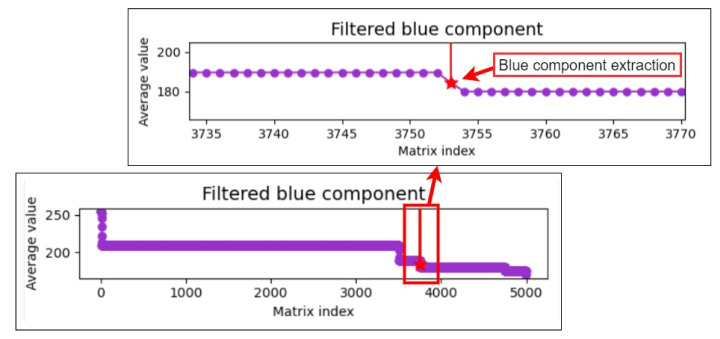
Upper limit extraction for the blue component.

**Figure 20:**
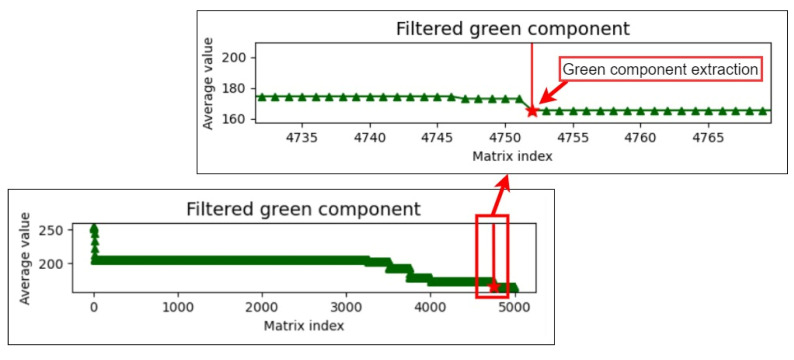
Upper limit extraction for the green component.

**Figure 21:**
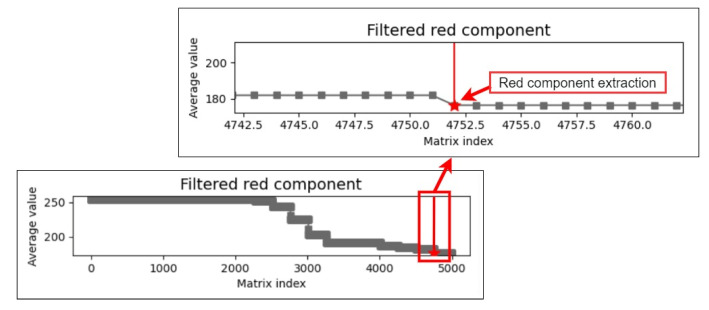
Upper limit extracted for the red component.

During the sequence detection process, the difference between the current and previous pieces of data is calculated. If this difference is greater than five, the final piece of data is extracted as the upper component limit as this piece may represent the threshold for differentiating the colors of the iris and pupil. Finally, the upper and lower component limits are added to the average. The mean of the aforementioned three values is used as the threshold for extracting the color of the pupil to reduce the effect of extreme values. Figure 19, Figure 20 and Figure 21 illustrate the extraction of the upper limits for the blue, green, and red components, respectively.

#### 3.1.4. Pupil Diameter Analysis

**Figure 22:**
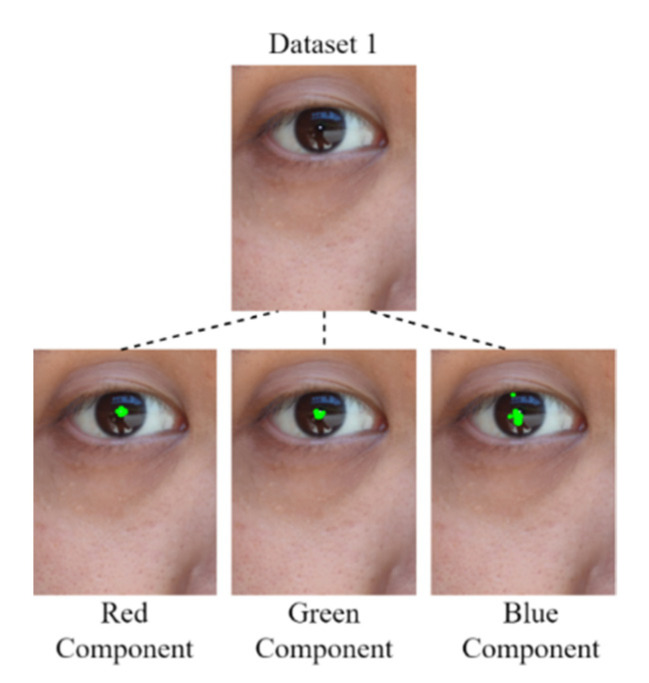
Image from Data Set 1.

**Figure 23:**
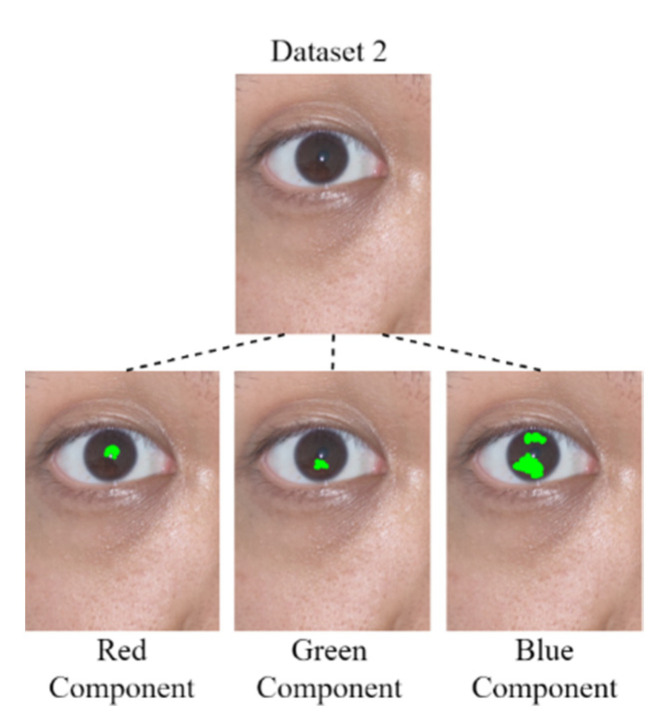
Image from Data Set 2.

**Figure 24:**
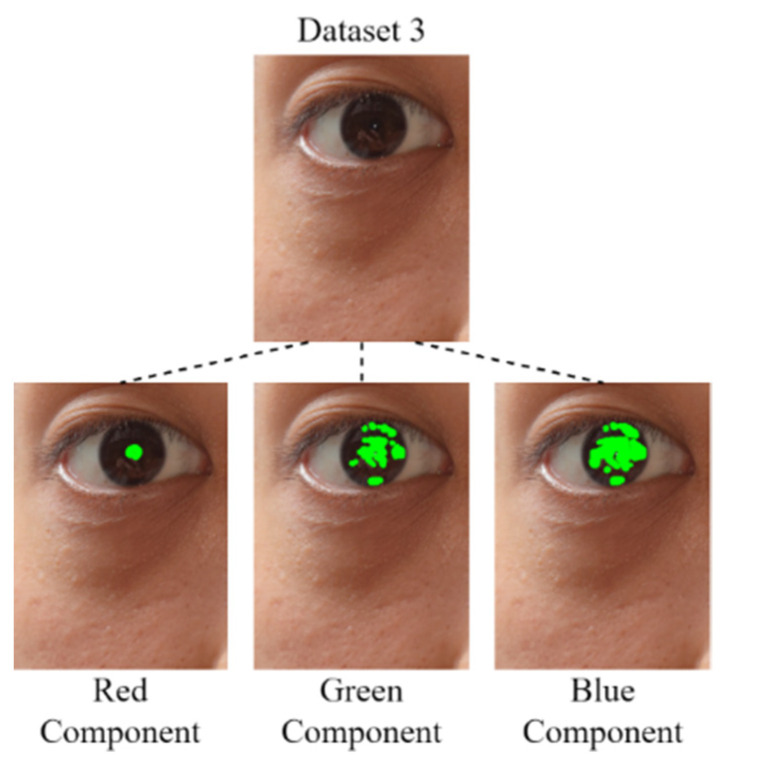
Image from Data Set 3.

**Figure 25:**
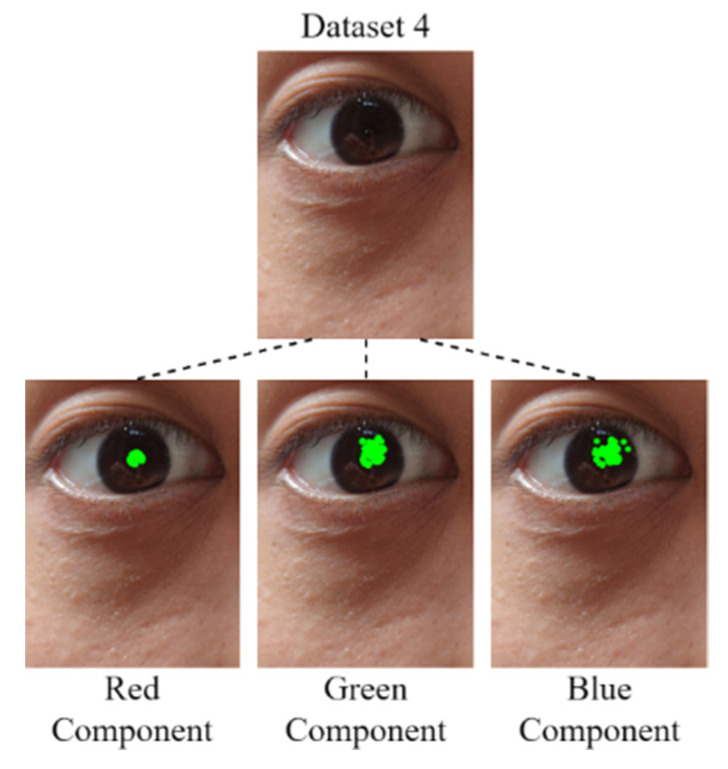
Image from Data Set 4.

After the derivation of the threshold for each color component by using the developed component threshold decision module, the RGB color channels are analyzed. In this study, four data sets were analyzed: Data Set 1, comprising an eye image captured for an Asian individual under outdoor sunlight; Data Set 2, comprising eye images captured for Asian individuals under outdoor sunlight; Data Set 3, comprising an eye image captured for an Asian individual under outdoor sunlight; and Data Set 4, comprising eye images captured for Asian individuals under outdoor sunlight. Images for which the mean color component was less than the threshold of each color component were derived for comparison with the original image. Figure 22, Figure 23, Figure 24 and Figure 25 depict eye pupils from Data Sets 1–4, respectively.

**Figure 26:**
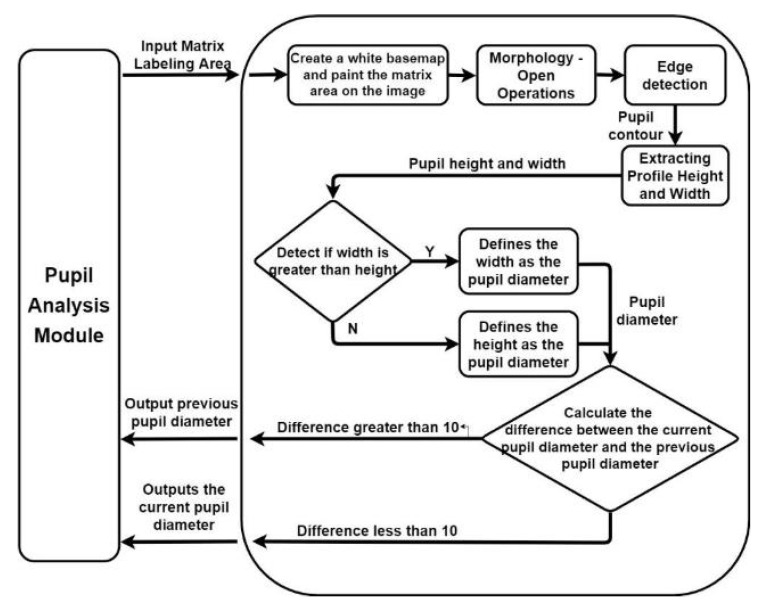
Architecture of the developed CCLPF-based pupil analysis module.

We identified the pupil areas in the images from Data Sets 1–4. The eye images taken in different outdoor light environments can be marked less than red the matrix area of the color component threshold; thus, the pupil position could be accurately determined. Therefore, we used this feature to conduct pupil diameter analysis. We designed a CCLPF-based pupil analysis module to extract the blocks marked by the matrix for analysis. Figure 26 depicts the architecture of this module.

**Figure 27:**
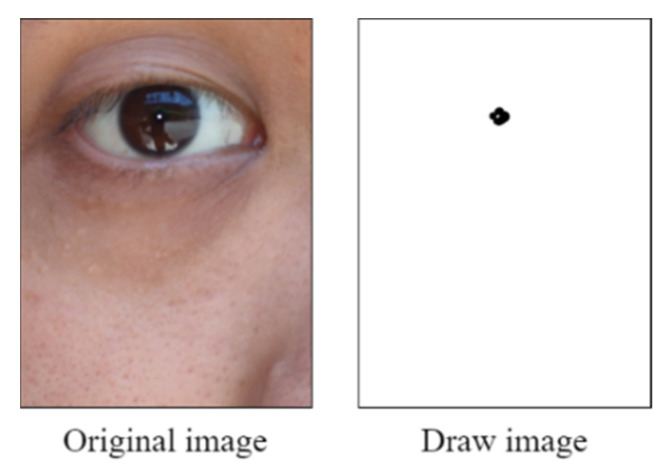
Matrix area below the red threshold.

We first created a blank image and plotted a matrix area below the red threshold. Figure 27 illustrates this matrix area. The middle area of the drawn blank image contained some white noise due to the reflection from the pupil.

**Figure 28:**
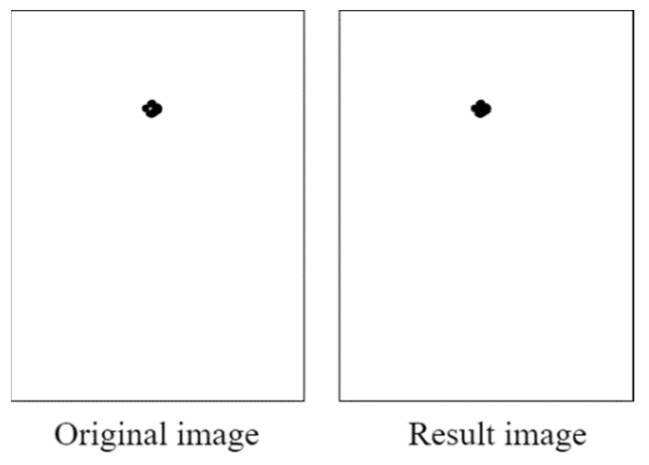
Image obtained after the open operation.

To filter out noise, the opening operation was conducted using 10 × 10 structural elements. Reflections (considered noise) were removed from the images. Figure 28 displays an image obtained after the opening operation. After an image was filtered through the opening operation, the pupil contour was extracted using the contour detection (findContours) function from the Python OpenCV library. This function is based on the topological structure described by Suzuki [16]. The pupil contour could be accurately extracted using the aforementioned function; thus, the pupil diameter data could be analyzed smoothly.

**Figure 29:**
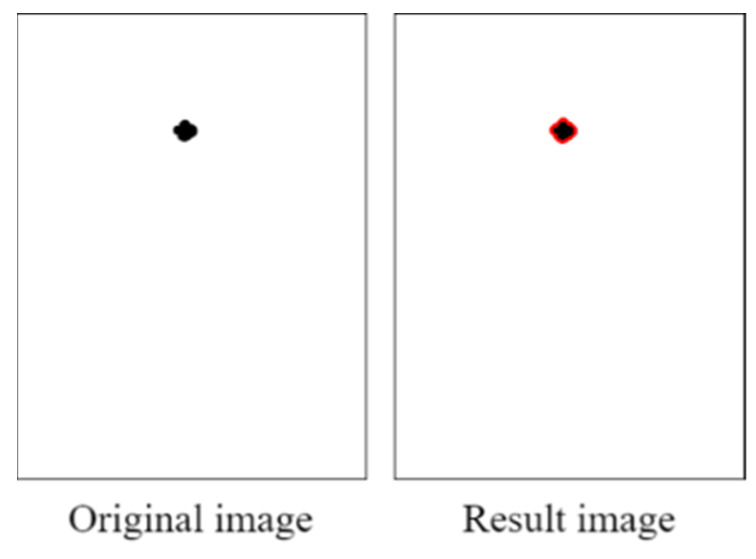
Results of the contour test.

Figure 29 displays the results of the contour test. The collected pupil data sets included some imperfect images; therefore, some imperfect pupil contours were obtained. To reduce the error caused by imperfect pupil contours, the maximum height and width of the pupil contours were used to determine the pupil diameters.

**Figure 30:**
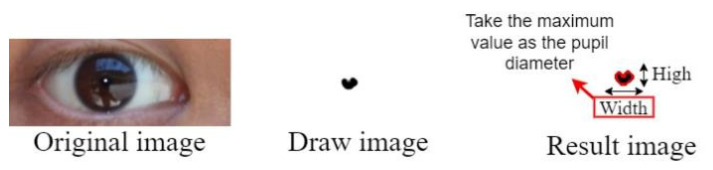
Imperfect pupil image.

**Figure 31:**
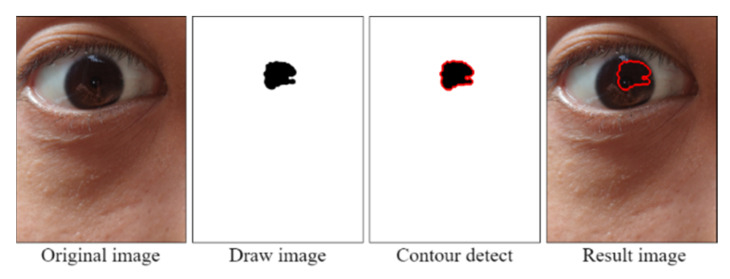
Analysis of inaccurate pupil diameter data for Data Set 4.

We used the rule-of-thumb principle to compare the current and previous pupil diameters. If the difference between these pupil diameters was higher than 10 pixels, the previous pupil diameter was used as the current pupil diameter to filter out inaccurate pupil diameter data. Figure 30 and Figure 31 illustrates the analysis of inaccurate pupil diameter data for Data Set 4.

#### 3.1.5. CCLPF Algorithm

**Figure 32:**
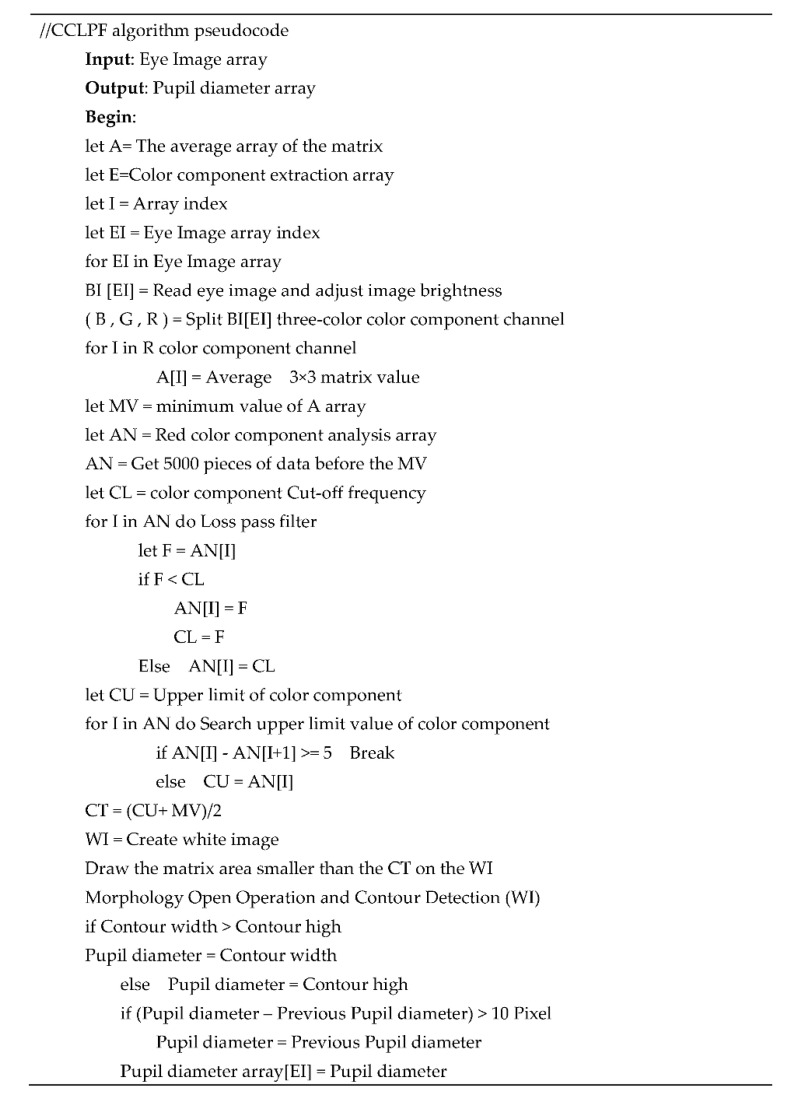
Pseudocode of the CCLPF algorithm.

The proposed CCLPF algorithm, whose pseudocode is displayed in Figure 32, conducts pupil diameter analysis by reading an imported image of the eyes. The imported image is adjusted (see lines 10 and 11). This image contains three color channels, and a 3 × 3 average matrix is derived for the red component in the image (see line 12). Noise is filtered through LPF (see line 17), and upper limit extraction is then performed for the red component (line 25). The threshold operation for the red component is executed (line 31). Finally, a new white image is created (line 32), and areas for which the average matrix is less than the threshold for the red component are derived. After the morphological operations, contour detection, and pupil diameter filtering, the pupil diameter is recorded in the array and then output.

The CCLPF algorithm can detect the eyes of Asian people in images captured under outdoor sunlight. By analyzing the components of each color channel, we found that the red component was correlated with the color of the pupils of Asian individuals in the aforementioned images. We compared the detection results of the CCLPF algorithm with those of the other algorithm [1].

### 3.2. PRSSM Algorithm

Color space conversion can be conducted to enhance the accuracy of pupil diameter analysis for images captured under different light sources. Images in the RGB color space can be transformed into the HSV color space through a simple transformation. The HSV color space represents the hue, saturation, and luminance of an image. We found that for images captured in outdoor environments, the pupil and iris of Asian individuals could be effectively differentiated through HSV color space conversion. In this study, the maximum error obtained in the classification of Data Set 5 using the PRSSM algorithm, which involves HSV conversion, was 20 pixels lower than that obtained in the same classification using the CCLPF algorithm. The PRSSM algorithm is described in detail in the following subsections.

#### 3.2.1. HSV Color Space Conversion

The RGB color space is represented using a linear combination of three highly correlated color components. Thus, to achieve continuous color transformation for an RGB image, the intensities of all three color components should be varied simultaneously. The three color components of an RGB image are closely related to image darkness. When the image darkness changes, variations occur in the intensities of these three components. No intuitive method exists for expressing these variations.

Accordingly, the RGB color space is suitable for the display of hardware systems, although not for image processing. The HSV color space is suitable for image processing, and the formula for converting the RGB color space into the HSV color space is as follows:

(1) $$\begin{array}{c} h=\{\;\begin{array}{c} 0\;\;\;\;\;\;\;\;,if\;\max=\min\;\\ 60\times \frac{g-b}{max-min}+0\;\;,if\;\max=r,g\geq b\\ 60\times \frac{g-b}{max-min}+360\;,if\;\max=r,g\leq b\\ 60\times \frac{b-r}{max-min}+120\;,if\;\max=g\\ 60\times \frac{b-r}{max-min}+240\;,if\;\max=b \end{array}\\ s=\{\;\begin{array}{c} 0\;\;\;\;\;\;\;\;,if\;\max=0\;\\ \frac{\max-min}{max}=1-\frac{min}{max}\;\;,\;otherwise \end{array}\\ v=max \end{array}$$

**Figure 33:**
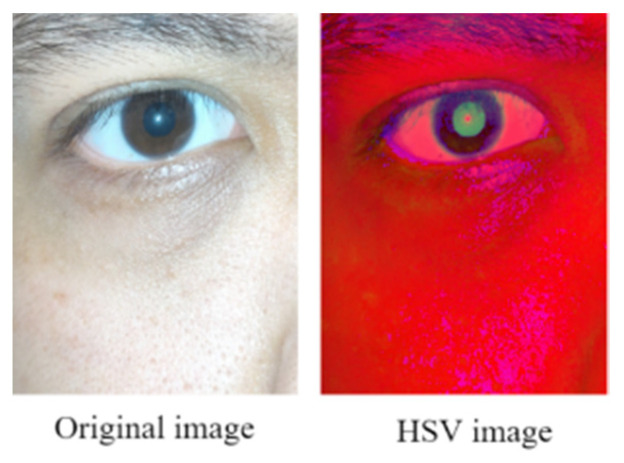
Original RGB image and corresponding image obtained after HSV color conversion.

The term *max* in Equation (1) indicates the highest intensity in the RGB color space, and *min* represents the lowest intensity in this space. Figure 33 displays the image obtained after conducting HSV color space conversion on a pupil image captured for an Asian individual in an indoor environment.

Figure 33 indicates that the color contrast between the iris and the pupil is considerably higher in the HSV image than in the original RGB image. Therefore, we should extract the distribution range of the pupil color in the HSV color space.

**Figure 34:**
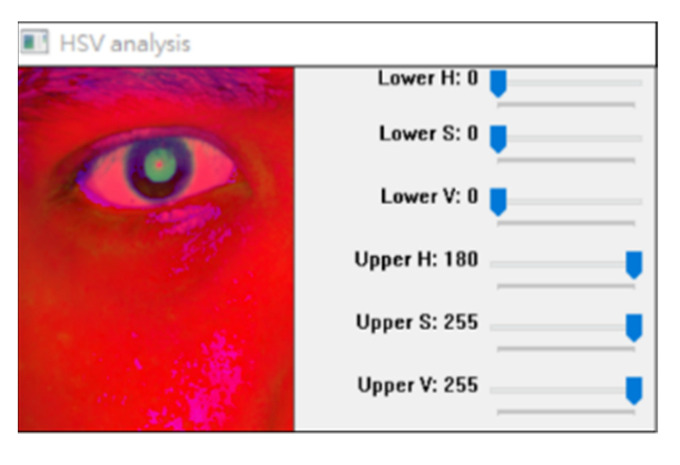
HSV color marker.

We designed an HSV analysis program to analyze the correlation between HSV color channels and the pupil color. Figure 34 depicts the interface of the aforementioned program. In this figure, the lower H, S and V represent the lowest hue, saturation, and luminosity thresholds, respectively; moreover, the upper H, S and V represent the highest hue, saturation, and luminosity thresholds, respectively.

**Figure 35:**
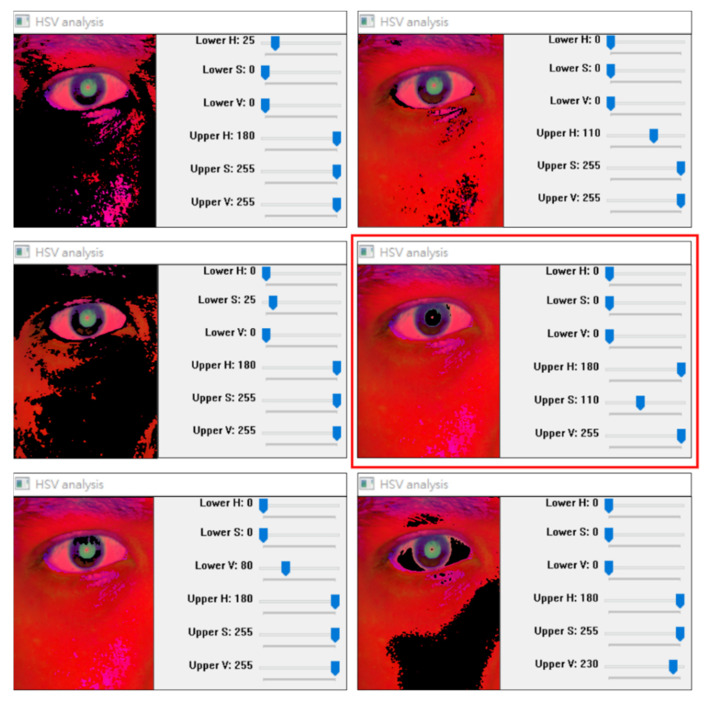
Adjustment of the color of each channel threshold mark.

We investigated the color marking phenomenon by fixing the threshold interval of two channels in the HSV color space and adjusting the upper and lower thresholds of the remaining channel (Figure 35). The lower H, S, and V thresholds in the red frame displayed in Figure 35 were set to zero. The upper H and V thresholds were set to 180 and 255, respectively. The upper S threshold obtained under the aforementioned conditions effectively indicated the pupil color range.

For the portion of outdoor light reflection, this paper uses HSV color space conversion to adjust color saturation in the image by using color space conversion. If the image has light reflections, we can still search for the contour position of the pupil through the color space conversion method. The lost pupil data will be corrected by using the algorithm of open and closed operation mentioned in this study.

The section on the test case is shown in Figure 34 through Figure 35 of this paper. Even if the light reflects, it does not affect the general position of the pupil, and the contours of the pupil can still be drawn.

#### 3.2.2. Adaptive Saturation Threshold Filtering

**Figure 36:**
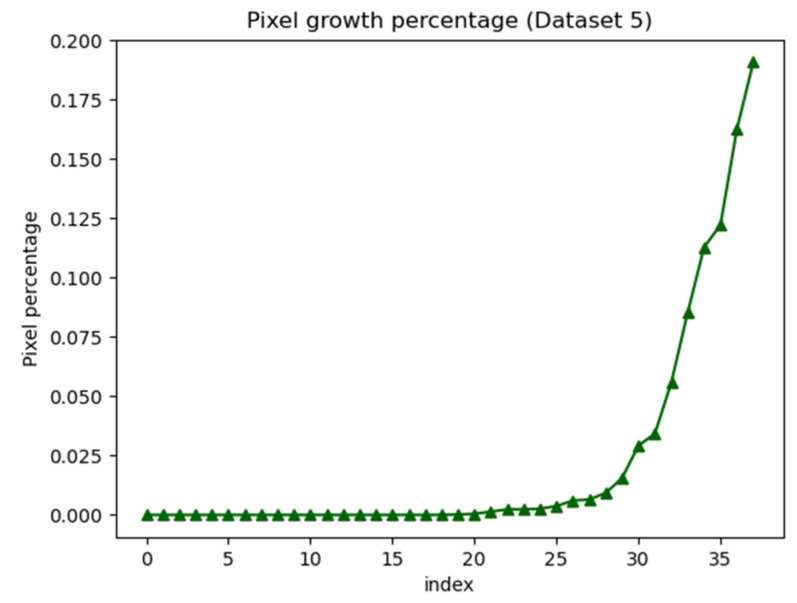
Graph of pixel growth ratio.

After the analysis of HSV color markers, the saturation in the HSV color space can be assessed. We can adjust the color saturation and then reduce the upper limit of the color saturation threshold by five each time to examine the increase in the pixel growth ratio. As shown in Equation (2), the pixel growth ratio can be derived as follows:

(2) $$Gr=\frac{\left(P_{n}-P_{n-1}\right)}{S}\;\;\;,\;if\;n>0$$

where *G_r_* denoted the pixel growth ratio; *P_n_* and *P_n −_*_1_ denote the numbers of pixels extracted in stages *n* and *n* − 1, respectively; and *S* denotes the total number of pixels. Figure 36 displays a graph of the ratio of pixel growth.

**Figure 37:**
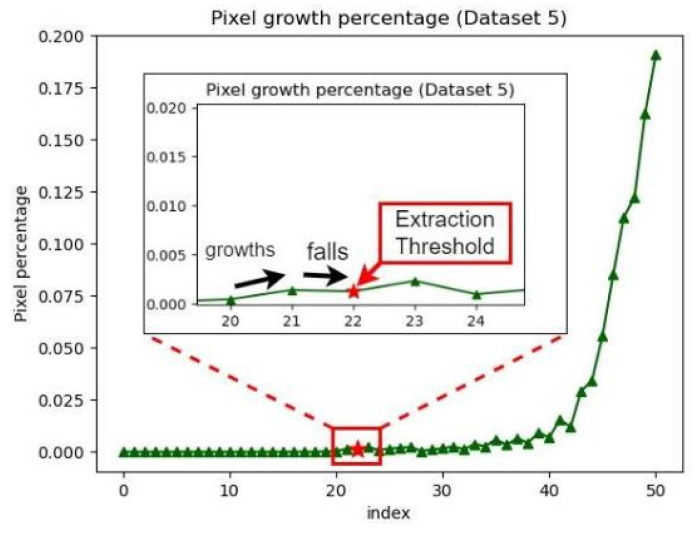
First decline in the pixel growth ratio.

In Figure 36, the *x*-axis represents the current index value, and the *y*-axis represents the pixel growth ratio. As the pupil color can be obtained by adjusting the saturation, the pixel growth ratio would first drop at the upper saturation threshold. Figure 37 illustrates the first decline in the pixel growth ratio.

**Figure 38:**
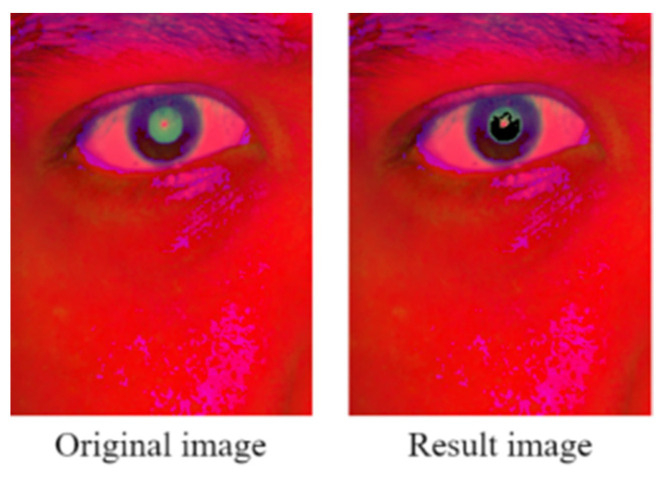
Pupil color distribution.

In this study, we varied the color saturation threshold and examined the corresponding first declines in the pixel growth ratio. We found that the first drop in the pixel growth ratio occurred at the upper saturation threshold. Figure 38 shows the pupil color distribution and the eye image obtained when the upper saturation threshold was used.

**Figure 39:**
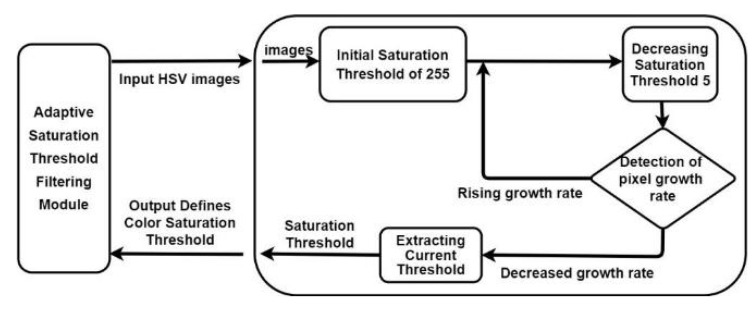
Developed module for adaptive saturation threshold filtering.

We designed a module for adaptive thresholding for extracting indoor artificial light from the first drop in the color saturation rate to effectively determine the pupil color distribution. Figure 39 depicts the aforementioned module.

The developed adaptive thresholding module first reads the input HSV image and initializes the upper saturation threshold of 255. For every five pixels, it then checks whether the current pixel growth ratio is less than the previous pixel growth ratio. If the current pixel growth ratio is higher than the previous pixel growth ratio, the module reduces the number of pixels until the current ratio is less than the previous ratio and then extracts and outputs the color saturation extraction threshold for the final decision.

#### 3.2.3. Pupil Diameter Analysis

**Figure 40:**
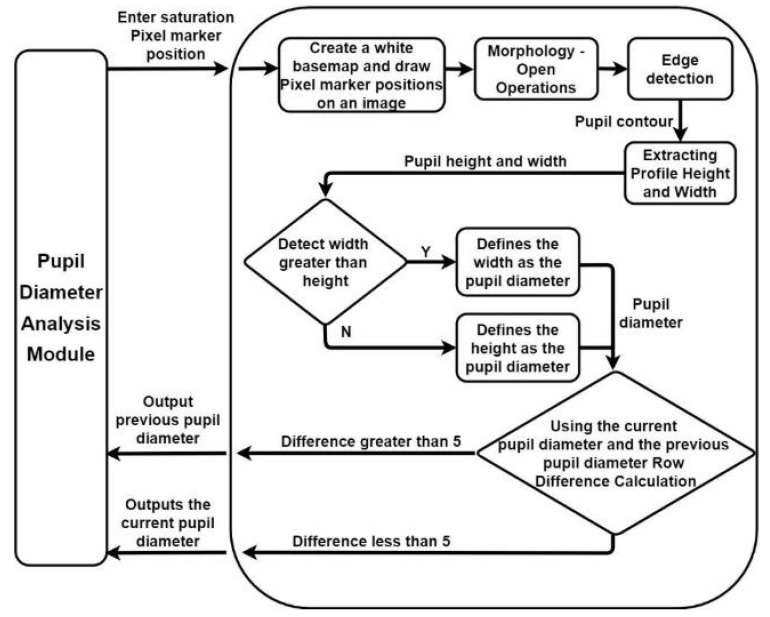
Developed pupil stateful recognition service mechanism (PRSSM)-based pupil diameter analysis module.

We used a pupil diameter analysis module based on the PRSSM algorithm to analyze the pupil diameter in images captured for Asian individuals under indoor artificial light conditions. The CCLPF algorithm defines a matrix area below the decision threshold of the red component on a blank image. By contrast, the PRSSM algorithm defines pixels in the HSV color space below the final decision on a blank image and decreases the comparison threshold for the final decision step to five pixels as this algorithm is more stable than the CCLPF algorithm. The PRSSM-based pupil diameter analysis module is illustrated in Figure 40.

**Figure 41:**
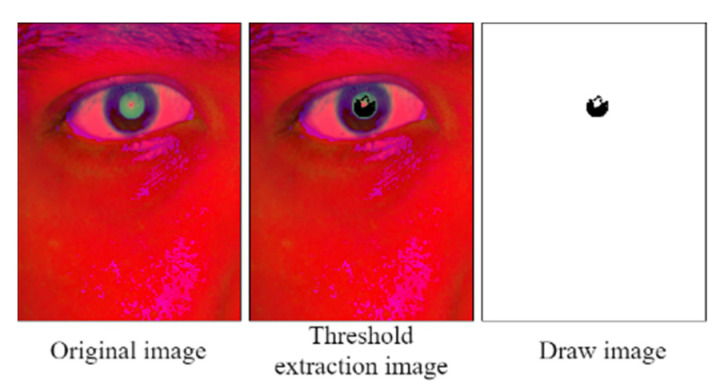
Pixel coordinate image obtained with the PRSSM-based pupil diameter analysis module.

The developed PRSSM-based pupil analysis module creates a blank image (similar to the CCLPF-based pupil analysis module) and defines a pixel below the final decision on the blank image. Figure 41 depicts the pixel coordinate image obtained with this module. A low level of white noise exists in this image due to the reflection of the pupil crystal.

**Figure 42:**
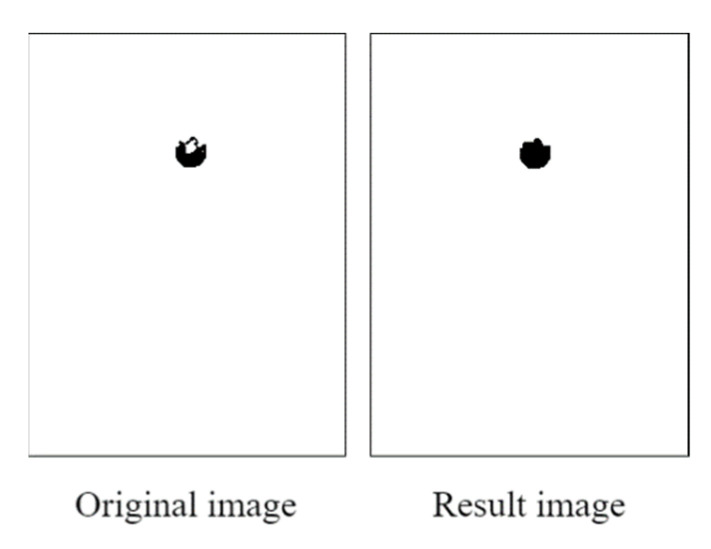
Image obtained with the PRSSM-based pupil diameter analysis module after the opening operation.

To filter out this noise, the developed PRSSM-based pupil analysis module uses the opening technique for filling in the drawn image. After the open operation is performed, the pupil contour is calculated using the findContours function from the Python OpenCV library, which allows for the smooth analysis of pupil diameter data. Figure 42 displays Image obtained with the PRSSM-based pupil diameter analysis module after the opening operation.

**Figure 43:**
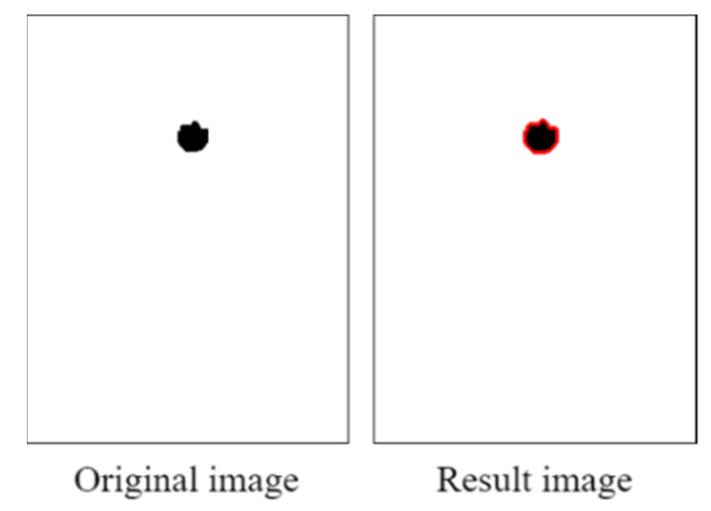
Results of the contour test obtained with the PRSSM-based pupil diameter analysis module.

Figure 43 displays the results of the contour tests obtained with the PRSSM-based pupil diameter analysis module. After contour detection, the maximum height and width of the contour are considered as the pupil diameter as the pupil marking in the input images is imperfect. The aforementioned step can reduce the error in pupil diameter analysis.

The current pupil diameter is compared with the previously analyzed pupil diameter in the CCLPF and PRSSM-based pupil diameter analysis modules. In the CCLPF-based module, if the error between the current and previous pupil diameters is higher than 10 pixels, the previous pupil diameter is used as the current pupil diameter. In the PRSSM-based module, the maximum permissible pupil diameter error is five pixels for images captured under indoor conditions. The developed PRSSM-based module can detect the pupil diameter through a stable process with low error rates.

#### 3.2.4. PRSSM Algorithm

**Figure 44:**
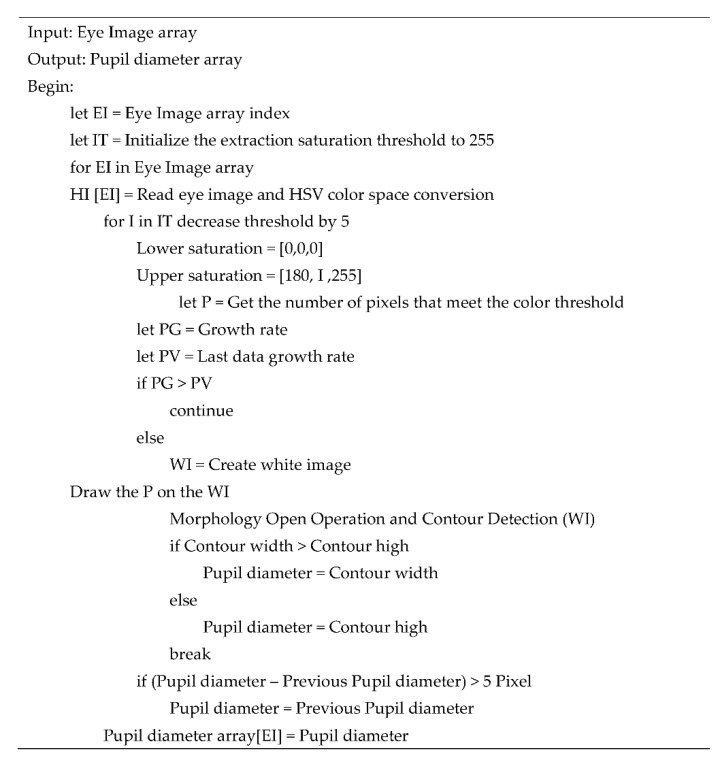
Pseudocode of the developed PRSSM algorithm.

The pseudocode of the developed PRSSM algorithm is presented in Figure 44.

The developed PRSSM algorithm performs pupil diameter analysis on an input eye image captured for an Asian person in an indoor environment. The input image is read and converted into the HSV color space (see line 7). Subsequently, the algorithm performs color saturation threshold extraction on the obtained HSV image (see line 8). It then calculates the growth rate of the extracted pixel. If the current growth rate is higher than the previous one, pixel extraction continues; however, if the current growth rate is less than the previous one, this threshold is used as the color saturation threshold, and a white image is created to depict the extracted pixel coordinates. After contour detection and pupil diameter filtering, the pupil diameter is recorded in an array, and the pupil diameter data set is output.

**Figure 45:**
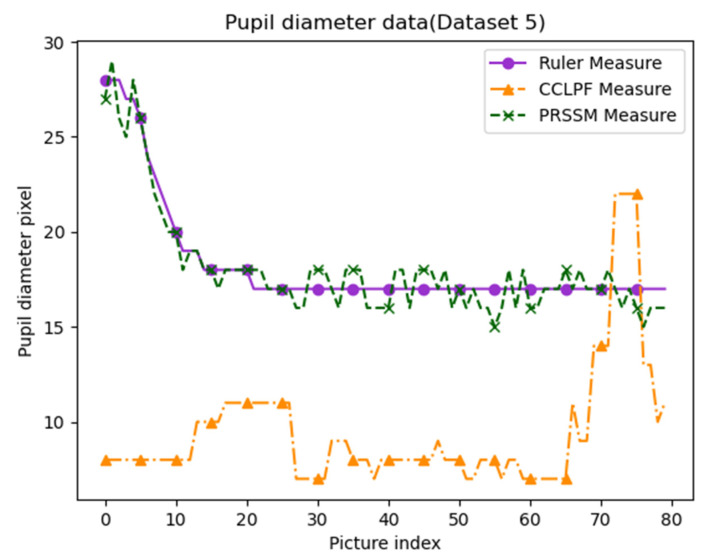
Pupil diameter graph for Data Set 5.

We executed the CCLPF and PRSSM algorithms on Data Set 5, which comprises 80 eye images captured for Asian people under indoor artificial light. These images have a resolution of 333 × 250 pixels and were captured using smartphones. For comparison, pupil diameter data that were obtained using a conventional ruler were included as the standard reference. Figure 45 displays a graph of the pupil diameter data obtained for Data Set 5. In this figure, the *x*-axis represents the picture index, and the *y*-axis represents the pupil diameter. The solid purple, dashed green, and dashed orange curves in Figure 45 represent the pupil diameter data obtained through the conventional ruler, using the PRSSM algorithm, and using the CCLPF algorithm, respectively. The results obtained with the PRSSM algorithm were considerably closer to the reference data than those obtained using the CCLPF algorithm. Thus, for images captured under indoor artificial light, the PRSSM algorithm can provide more accurate pupil diameter results compared with the CCLPF algorithm.

**Figure 46:**
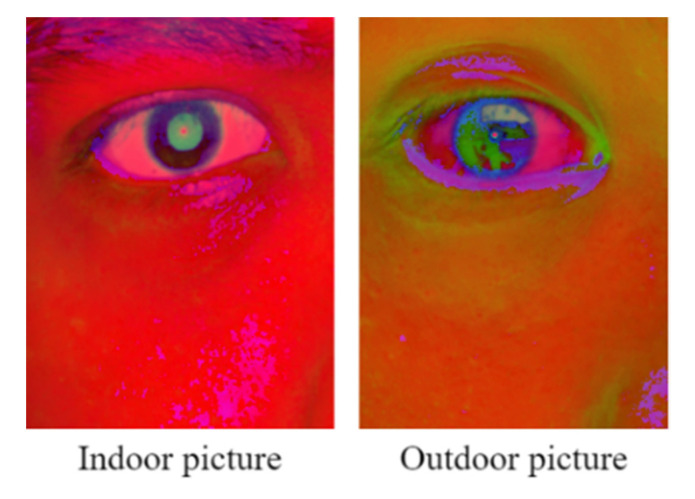
HSV eye images captured for Asian people in indoor and outdoor environments.

However, the PRSSM algorithm is unsuitable for determining the pupil diameter from images captured under outdoor sunlight due to the presence of different ambient light sources in outdoor environments. The color contrast between the pupil and iris of an Asian individual in HSV images captured under outdoor environments is lower than that in HSV images captured indoors. Figure 46 depicts HSV eye images captured for Asian people in indoor and outdoor environments.

The CCLPF algorithm can effectively determine the pupil diameter in images captured under outdoor sunlight by analyzing the characteristics of the red component threshold and extracting the matrix area below this threshold. The PRSSM algorithm can effectively determine the pupil diameter by converting an RGB image into an HSV image through adaptive thresholding and the extraction of pixel coordinate points under the color saturation threshold. Experiments were performed to compare these two algorithms with the other algorithm [1].

## 4. Results

The developed CCLPF and PRSSM algorithms were compared with the other algorithm [1]. In [1], the RGB channels are converted into HSV channels, and grayscaling and histogram equalization are applied.

**Figure 47:**
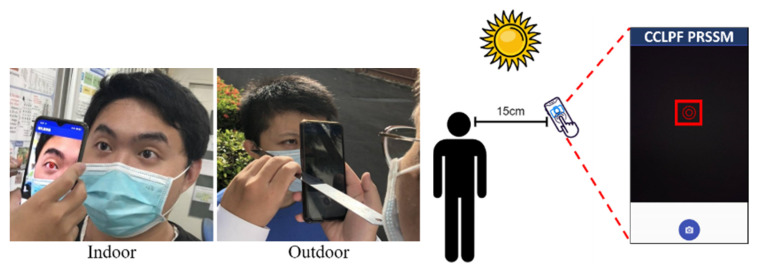
Schematic of the experiment data set.

The fourteen data sets used in this study contained images of fourteen Asian individuals, of whom seven were men and seven were women (Table 3). Figure 47 shows a schematic of the experimental data set. During our experiment, these individuals were invited to visit fourteen sites; Table 4 indicates the locations at which the images in the data sets were captured. Each data set contained 80 images of the eyes of the individuals. These images had a resolution of 333 × 250 pixels and were captured quickly using a smartphone in different light environments. All images were captured with the smartphone’s flash light activated. We compared the experimental results obtained for the 14 adopted data sets using the CCLPF and PRSSM algorithms with those obtained using the other algorithm [1]. The programing language used in this study was Python (version 3.8.3), and the adopted hardware was an Intel Core i7-8550U CPU with a 1.80- and 1.99-GHz processor and 8.00 GB RAM. All the smartphones used for capturing the images hosted the Android (version 10) operating system.

One image was selected from each of the data sets (Data Sets 1–14) and input into the other algorithm [1]. The intensities of the blue and red components were manipulated to obtain a grayscale version of the input image, thus effectively enhancing the iris detection effect. The general grayscale operation involved obtaining a grayscale image by multiplying the intensities of the red, blue, and green components by different values. This operation can be expressed as follows:

*Gray* = *R* × 0.299 + *G* × 0.587 + *B* × 0.114 (3)

where *R*, *G*, and *B* represent the intensities of the red, blue, and green components, respectively, and *Gray* represents the grayscale intensity. We replaced the intensity of the green component in Equation (3) with the intensity of the blue component to obtain the following grayscaling formula:

*Gray* = *R* × 0.299 + *B* × 0.587 + *B* × 0.114 (4)

**Figure 48:**
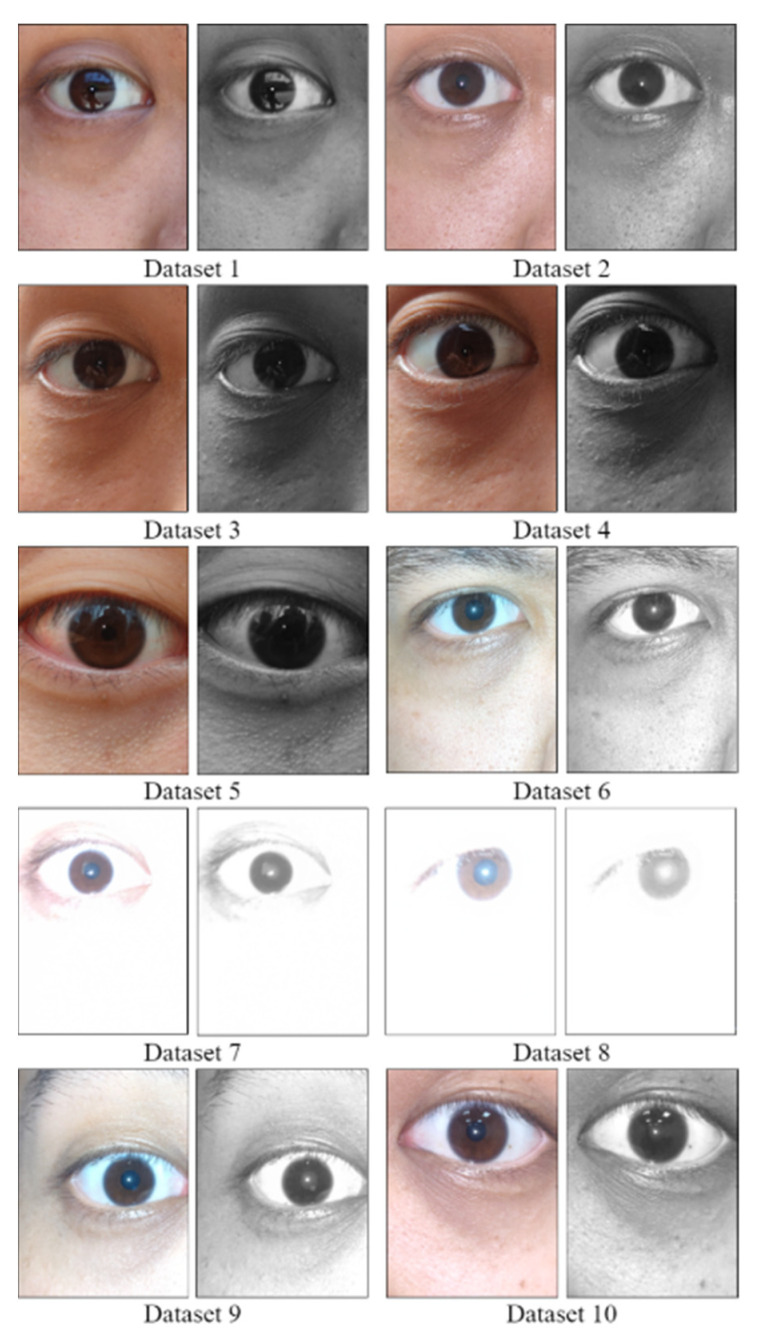
Results obtained after grayscaling for each data set.

As shown in Equation (4), this formula was used for image grayscaling. Figure 48 displays the grayscaling results for each data set.

**Figure 49:**
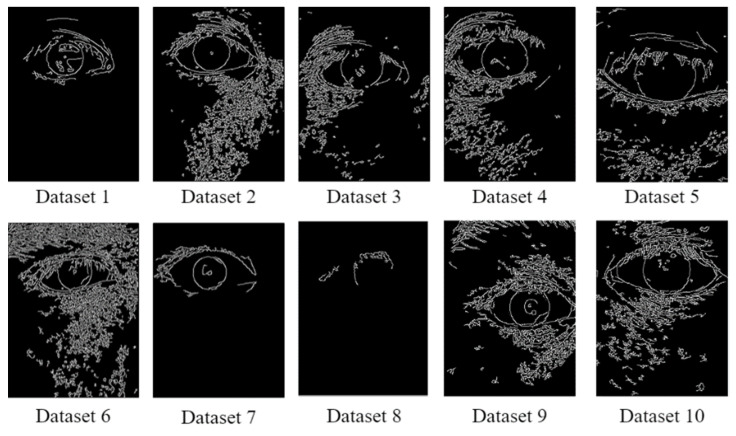
Results obtained for each data set through the Canny edge detector.

After grayscale images were obtained for each data set, Canny edge detection was executed on these images by using the Canny function in Python OpenCV. The literature does not mention suitable threshold values for Canny edge detection; therefore, we set 50 and 150 as the lower and upper thresholds, respectively. Figure 49 depicts the Canny edge detection results obtained for each data set.

**Figure 50:**
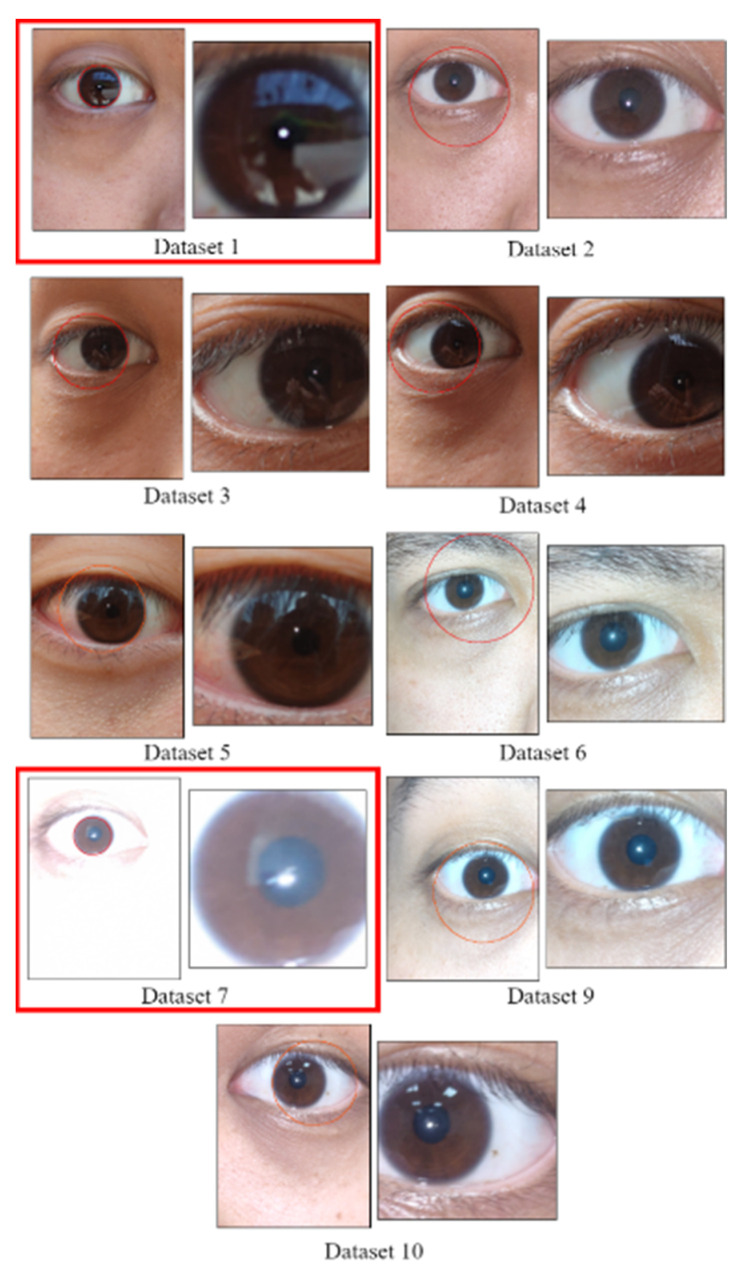
Results of the Hough circle test for each data set.

We used the HoughCircles function in Python OpenCV for circle detection. As the iris could be accurately identified only for Data Sets 1 and 7, we compared the performance of the other algorithm [1] with that of the CCLPF and PRSSM algorithms for these data sets only. Figure 50 illustrates the Hough detection results for each data set. In this figure, the images in the red frame (i.e., Data Sets 1 and 7) were adopted for further analysis; the results for Data Set 8 are not shown in this figure as the iris position could not be determined using the HoughCircles function.

**Figure 51:**
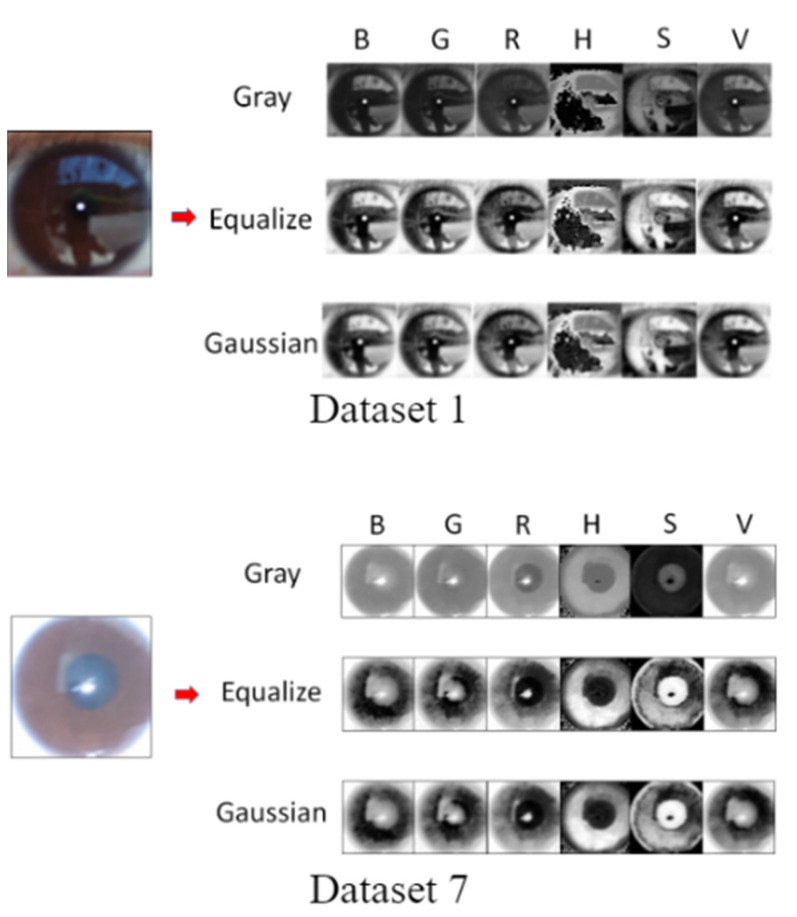
Iris color range obtained after each processing stage for Data Sets 1 and 7.

After comparing the results obtained with the three investigated algorithms, we used the proposed method to perform single-channel grayscaling, histogram equalization, and Gaussian fuzzy calculation for each RGB and HSV channel. Figure 51 shows the iris color range obtained after each processing stage for Data Sets 1 and 7.

**Figure 52:**
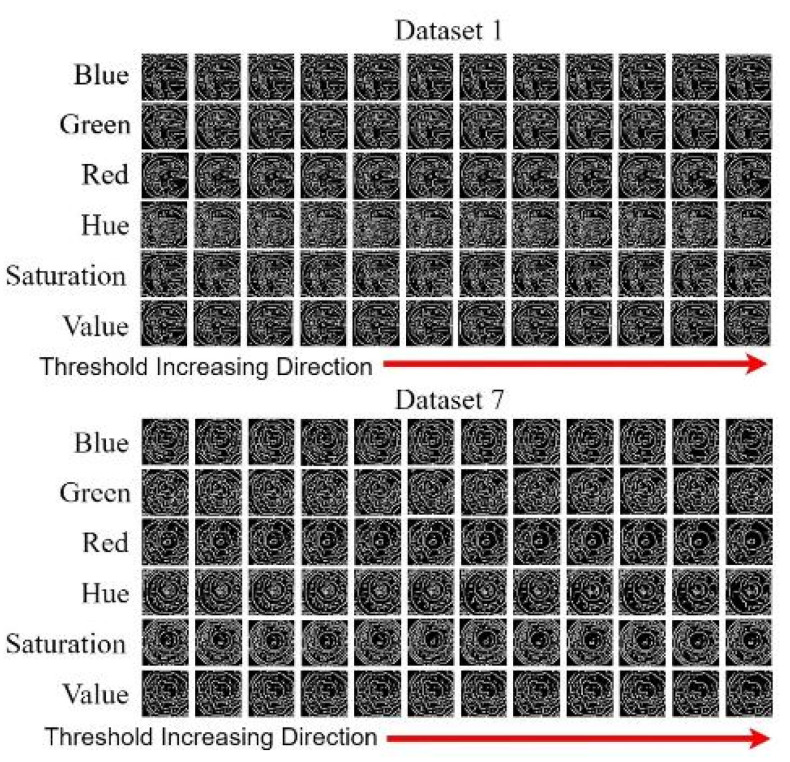
Results obtained after Canny edge detection.

**Figure 53:**
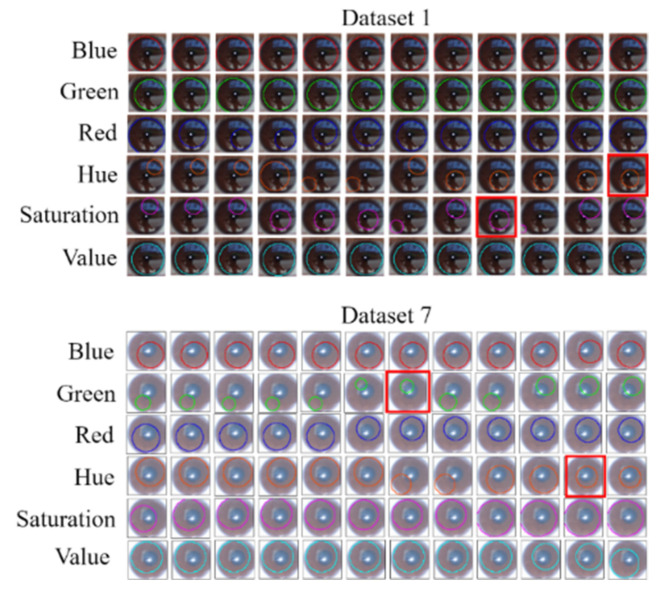
Results of the Hough circle test.

When an iris image is subjected to Gaussian blurring, Canny edge detection based on Hough circles must be performed to automatically increase the detection threshold and obtain the pupil contour. The detection threshold should be increased by 10–70 in increments of five. As two thresholds are used in Canny edge detection, we defined a lower threshold as well as an upper threshold that was twice the lower threshold to achieve optimal results. Pupil contour extraction was performed for Data Sets 1 and 7. The extracted pupil profile indicated the type of channel and the required Canny threshold. Figure 52 displays the Canny edge detection results obtained for each color channel for the two data sets. Figure 53 depicts the results of the Hough circle test for the two data sets. This test was conducted after the execution of Canny edge detection.

In Figure 53, the results obtained using the other algorithm [1] are indicated in the red frames. For Data Set 1, we extracted pupils through Hough circle detection by using the hue and saturation channels and by setting the upper and lower thresholds for Canny edge detection to 65 and 50, respectively. For Data Set 7, we extracted pupils through Hough circle detection by using the green channel along with the hue channel and by setting the upper and lower thresholds for Canny edge detection to 60 and 40, respectively.

On the basis of the aforementioned analysis, we determined the pupil diameters for the images from Data Sets 1 and 7. We compared the results obtained for Data Set 1 using the other algorithm [1] with those obtained for this data set using the CCLPF algorithm. We also compared the results obtained for Data Set 7 using the other algorithm, [1], with those obtained for this data set using the PRSSM algorithm.

**Figure 54:**
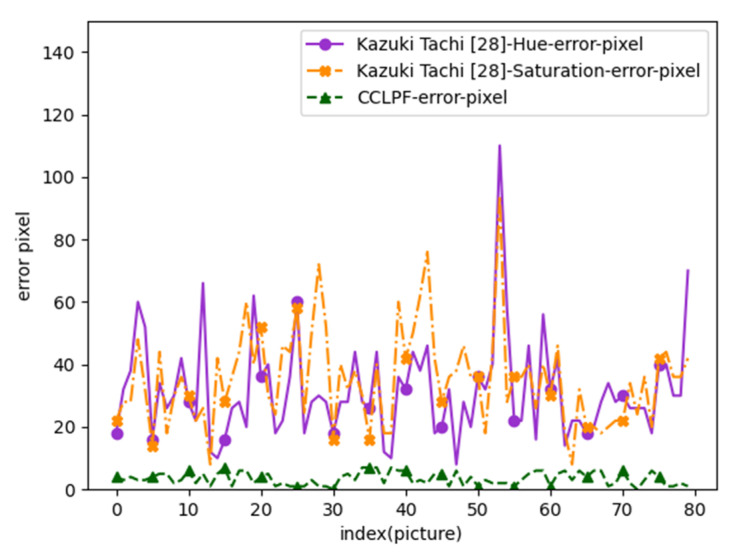
Measurement errors for Data Set 1.

Pupil diameters measured using a traditional ruler were used as the reference. For pupil diameter measurement using the other algorithm [1] for Data Set 1, the hue and saturation channels were used, and the upper and lower thresholds for Canny edge detection were set to 65 and 50, respectively. The measurement results obtained using this algorithm and those obtained using the CCLPF algorithm were compared with the reference data. Figure 54 displays the measurement errors for Data Set 1.

In Figure 54, the *x*-axis represents the image index, and the *y*-axis represents the errors between pupil diameter measurements obtained with a traditional ruler and the pupil diameter values obtained with the other algorithm [1] and the CCLPF algorithm. The error curve for the CCLPF algorithm was considerably lower and flatter than that for the other algorithm [1]. Thus, the CCLPF algorithm had higher stability and accuracy than did the other algorithm [1] for Data Set 1.

**Figure 55:**
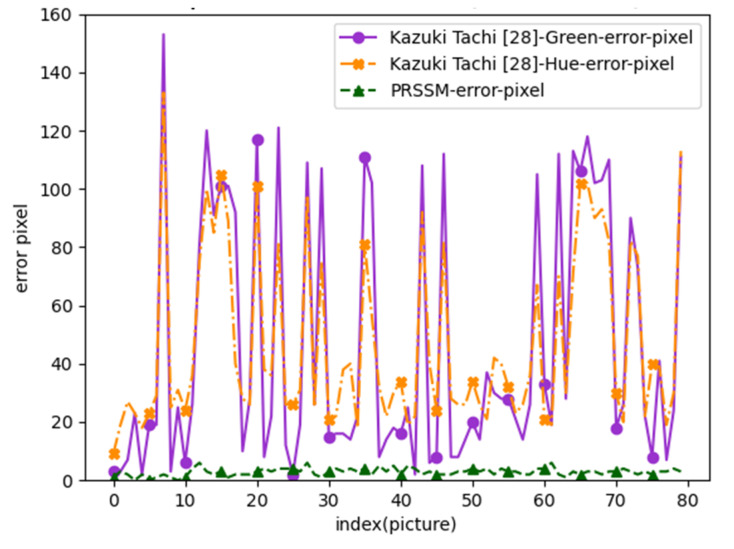
Measurement errors for Data Set 7.

Pupil diameters measured using a traditional ruler were also used as the reference. For pupil diameter measurement using the other algorithm [1] for Data Set 7, the green and hue channels were used, and the upper and lower thresholds for Canny edge detection were set to 50 and 40, respectively. The measurement results obtained using this algorithm and those obtained using the PRSSM algorithm were compared with the reference data. Figure 55 depicts the measurement errors for Data Set 7.

In Figure 55, the *x*-axis represents the image index, and the *y*-axis represents the errors between pupil diameter measurements obtained with the traditional ruler and the pupil diameter values obtained using the other algorithm [1] and the PRSSM algorithm for Data Set 7. The error curve for the PRSSM algorithm was lower and flatter than that of the other algorithm [1] for Data Set 7. Thus, the PRSSM algorithm had higher stability and accuracy than the other algorithm [1] for Data Set 7. We also compared the other algorithm [1] with the developed CCLPF and PRSSM algorithms. The CCLPF and PRSSM algorithms achieved higher accuracy rates than the other algorithm [1] for Data Sets 1 and 7, respectively.

**Figure 56:**
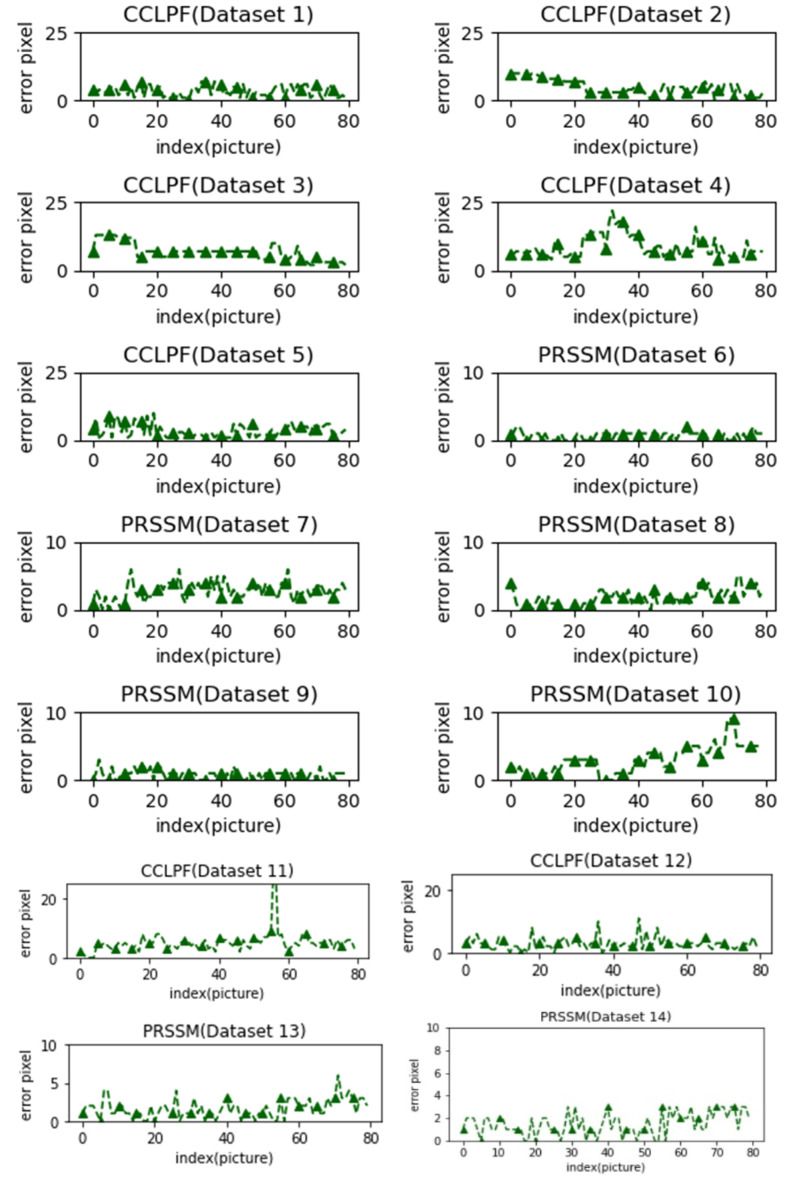
Pupil diameter errors for the PRSSM and CCLPF algorithms for the 10 data sets.

The pupil diameter data obtained for Data Sets 1–14 by using the traditional ruler methods were compared with those obtained using the CCLPF (for Data Sets 1–5 and 11–12) and PRSSM (for Data Sets 6–10 and 13–14) algorithms. Figure 56 displays the measurement errors for the PRSSM and CCLPF algorithms for different data sets.

As displayed in Figure 56, for Data Sets 1–5 and 11–12, the difference between the pupil diameters obtained using the CCLPF algorithm and those obtained through ruler measurements was less than 25 pixels. For Data Sets 6–10 and 13–14, the difference between the pupil diameters obtained using the PRSSM algorithm and those obtained through ruler measurements was less than 10 pixels.

**Figure 57:**
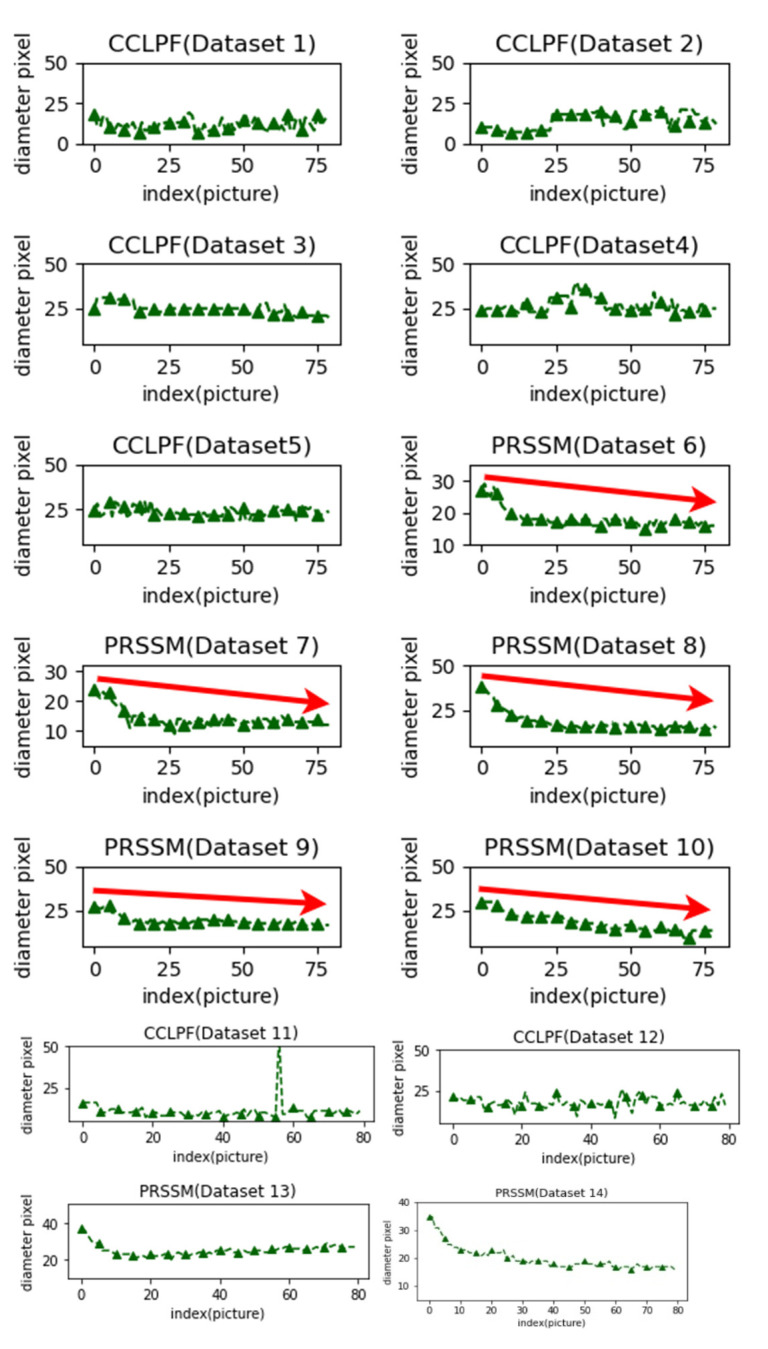
Measured pupil diameter data.

A previous study revealed that pupil diameter detection results could indicate pupil constriction. Accordingly, we used Data Sets 1–14 to explore changes in pupil size. We observe changes in pupil size between images captured in outdoor environments and those captured in indoor environments. This is due to the fact that in outdoor environments, the light prompted individuals to constrict their pupils, whereas in indoor environments, the relatively strong illuminance caused by the camera flash prompted the individuals to further constrict their pupils. Figure 57 displays a graph of the measured pupil diameter data for Data Sets 1–14.

The measured pupil diameter data for Data Sets 1–5 and 11–12, which was comprised of images captured outdoors, did not indicate noticeable constriction. However, the measured pupil diameter data for Data Sets 6–10 and 13–14, which were captured indoors, indicated clear pupil constriction. The PRSSM algorithm could effectively detect pupil constriction.

## 5. Discussions

We looked for the pupils of white people in most of the public data sets. Experimental analysis found that the pupil pattern is different from that of beige people, so the method we propose can analyze the pupil images of beige people. Color morphology analysis is needed to achieve a high accuracy rate.

We have developed a fast method that can be applied to universal mobile devices and can perform and check for pupil changes in general mobile devices, since most of the users are doctors. In most cases, this can be used in a space environment or in an environment without an internet connection.

In iris and pupil identification, infrared cameras are usually employed to capture eye images. The pupil position can be clearly identified in eye images captured using an infrared camera. However, most camera systems capture images using the RGB color channel. The eyes and iris of Asian individuals are usually brown or black. Such similar colors make it difficult to analyze pupil images captured for Asian individuals under natural and artificial light sources. Accordingly, the components of the RGB color space should be analyzed and converted to the HSV color space for pupil detection in images captured for Asian individuals.

Among the other methods we compare, we have compared the pupil with the only algorithm of the same type of algorithm applied to beige people and found that our proposed method has a higher accuracy rate and no drift problem. Since most of the methods currently require specific medical devices, we have developed a mobile device that is suitable for universal use, is fast, can be performed on general mobile devices and can check for pupil changes. We also compared the other algorithm [1] with the developed CCLPF and PRSSM algorithms. The CCLPF and PRSSM algorithms achieved higher accuracy rates than the other algorithm [1] for Data Sets 1 and 7, respectively. 

This paper contributes to the research literature in four major areas: (1) We propose the PRSSM algorithm and CCLPF algorithm, which can be used to analyze Asian pupil diameter under indoor artificial light and outdoor sunlight; (2) Solving the difficulty of analyzing the pupil diameter in Asians with similar color to iris; (3) We develop a universal mobile device that can be performed and checked for pupil changes on a general mobile device; 4) Improve recognition, application penetration and execution speed in different environments to realize these goals.

## 6. Conclusions

We developed the PRSSM and CCLPF algorithms for pupil diameter analysis on images captured under indoor artificial light and outdoor natural light, respectively. HSV color space conversion can effectively increase the contrast between the pupil and the iris in images captured in indoor environments with artificial light sources. The developed PRSSM algorithm was used to detect the pupil in eye images that were captured indoors in an Asian population. The relevant experimental results indicate that the PRSSM algorithm had high accuracy and stability in the aforementioned detection, with the error between the pupil diameter data obtained through conventional ruler measurements and with the PRSSM algorithm being less than 10 pixels.

The intensity of the red component of RGB images was found to be highly correlated with the color of the pupils in the Asian population. Therefore, an LPF algorithm was designed to detect the pupils of Asian individuals in images captured in outdoor environments under natural sunlight. The error between the pupil diameter data measured through conventional ruler measurements and data measured using the CCLPF algorithm for outdoor environments was less than 25 pixels; thus, the measurement accuracy and stability of the CCLPF algorithm were verified.

We develop a mobile device that can be used for universal use and can be performed and checked for pupil changes on a general mobile device. There is a set limit on the mobile device; you need to set the distance between the mobile device and the pupil first. However, we experimented with the flash brightness of each mobile device and found that it is slightly different on each device, which may affect the identification results, although this should only have a slight impact on the actual experiment.

## Figures and Tables

**Table 1:** Abbreviations.

Full Title	Abbreviations
Pupil stateful recognition service mechanism	PRSSM
Color component low-pass filtering	CCLPF
Hue Saturation Value	HSV
Low pass filtering	LPF

**Table 2:** Illuminance in different environments [19].

Environment	Exposure value (EV)
Under the hot sun	16
Under a cloudy sky	15
Mappings	14
In a reading room	13
In a baseball field at night	12
In an office or a classroom	12
Under street lights	8–11
Under a full moon	8–9
Under starlight	8

**Table 3:** Information on the data sets used in this study.

Data set	Participant	Age (Years)	Gender	Occupation
Data Set 1	A	18	Female	Student
Data Set 2	B	21	Female	Student
Data Set 3	C	17	Female	Student
Data Set 4	D	24	Male	Student
Data Set 5	E	23	Male	Student
Data Set 6	F	30	Male	Technology industry
Data Set 7	G	22	Female	Student
Data Set 8	H	18	Male	Student
Data Set 9	I	20	Male	Student
Data Set 10	J	28	Female	Service industry
Data Set 11	K	53	Male	woodworker
Data Set 12	L	51	Female	hairdresser
Data Set 13	M	21	Female	Student
Data Set 14	N	40	Male	IT Engineer

**Table 4:** Sources of each experimental data set.

Data Set	Source Of The Data Set
Data Set 1	Outdoor sunshine riding downstairs shooting (1)
Data Set 2	Outdoor sunshine riding downstairs shooting (2)
Data Set 3	Captured outdoor under natural sunlight (1)
Data Set 4	Captured outdoor under natural sunlight (2)
Data Set 5	Captured outdoor under natural sunlight (3)
Data Set 6	Captured in an office room
Data Set 7	Captured in a shady warehouse
Data Set 8	Captured under faint orange light in a washroom
Data Set 9	Captured in a bedroom
Data Set 10	Captured in a living room (1)
Data Set 11	Captured outdoor under natural sunlight (4)
Data Set 12	Captured outdoor under natural sunlight (5)
Data Set 13	Captured in a living room (2)
Data Set 14	Captured in a living room (3)

## Data Availability

Not applicable.
